# PredictSNP: Robust and Accurate Consensus Classifier for Prediction of Disease-Related Mutations

**DOI:** 10.1371/journal.pcbi.1003440

**Published:** 2014-01-16

**Authors:** Jaroslav Bendl, Jan Stourac, Ondrej Salanda, Antonin Pavelka, Eric D. Wieben, Jaroslav Zendulka, Jan Brezovsky, Jiri Damborsky

**Affiliations:** 1 Loschmidt Laboratories, Department of Experimental Biology and Research Centre for Toxic Compounds in the Environment, Faculty of Science, Masaryk University, Brno, Czech Republic; 2 Department of Information Systems, Faculty of Information Technology, Brno University of Technology, Brno, Czech Republic; 3 Center of Biomolecular and Cellular Engineering, International Centre for Clinical Research, St. Anne's University Hospital Brno, Brno, Czech Republic; 4 Department of Biochemistry and Molecular Biology, Mayo Clinic, Rochester, New York, United States of America; University of Canterbury, New Zealand

## Abstract

Single nucleotide variants represent a prevalent form of genetic variation. Mutations in the coding regions are frequently associated with the development of various genetic diseases. Computational tools for the prediction of the effects of mutations on protein function are very important for analysis of single nucleotide variants and their prioritization for experimental characterization. Many computational tools are already widely employed for this purpose. Unfortunately, their comparison and further improvement is hindered by large overlaps between the training datasets and benchmark datasets, which lead to biased and overly optimistic reported performances. In this study, we have constructed three independent datasets by removing all duplicities, inconsistencies and mutations previously used in the training of evaluated tools. The benchmark dataset containing over 43,000 mutations was employed for the unbiased evaluation of eight established prediction tools: MAPP, nsSNPAnalyzer, PANTHER, PhD-SNP, PolyPhen-1, PolyPhen-2, SIFT and SNAP. The six best performing tools were combined into a consensus classifier PredictSNP, resulting into significantly improved prediction performance, and at the same time returned results for all mutations, confirming that consensus prediction represents an accurate and robust alternative to the predictions delivered by individual tools. A user-friendly web interface enables easy access to all eight prediction tools, the consensus classifier PredictSNP and annotations from the Protein Mutant Database and the UniProt database. The web server and the datasets are freely available to the academic community at http://loschmidt.chemi.muni.cz/predictsnp.

This is a *PLOS Computational Biology* Software Article

## Introduction

The single nucleotide variants (SNVs) are the most frequent type of genetic variation in humans, responsible for almost 90% of known sequence differences [1], [2]. Although many of these changes are neutral, some variants do affect gene expression or the function of the translated proteins [3], [4]. Such SNVs often have dramatic phenotypic consequences leading to the development of various diseases [5]. Approximately half of the known disease-related mutations stems from non-synonymous SNVs, manifested as amino acid mutations [6], [7]. Although it is extremely important to uncover the links between SNVs and associated diseases, it is difficult to distinguish pathogenic substitutions from those that are functionally neutral by any experimental assay due to rapid growth of the number of known SNVs [8], [9]. Therefore, computational prediction tools became valuable for the initial analysis of SNVs and their prioritization for experimental characterization.

There are many computational tools for prediction of the effects of amino acid substitution on protein function, e.g., MutPred [10], nsSNPAnalyzer [11], PolyPhen-1 (PPH-1) [12], PolyPhen-2 (PPH-2) [13], SNAP [14], MAPP [15], PANTHER [16], PhD-SNP [17], SIFT [18] and SNPs&GO [19]. Most of these tools are designed to predict whether a particular substitution is neutral or deleterious, based on various parameters derived from the evolutionary, physico-chemical or structural characteristics [20], [21]. These tools mainly employ machine learning methods to derive their decision rules based on a training datasets of annotated mutations. Since overlaps between the training datasets of evaluated tools and the benchmark datasets have been shown to result in illegitimately high performance estimates of such tools [22], [23], it is of the utmost importance to carry out any comparisons on fully independent datasets [24], [25]. Variability of the training datasets utilized by individual prediction tools coupled with the public inaccessibility of these datasets represent a serious obstacle to unbiased comparison of predictive power of the tools [21]. Since individual prediction tools have been developed using different: (i) training datasets, (ii) machine learning methods and (iii) underlying principles, it is generally believed that some of them can be combined to give a single consensus prediction with improved accuracy [26]. Recent examples of consensus tools are CONDEL [27], PON-P [28] and Meta-SNP [29], all of which reported improved performance over individual integrated tools.

In this paper, we constructed three fully independent datasets, one benchmark and two testing datasets, suitable for assessment of the performance of eight selected prediction tools in an unbiased manner. We then combined six best performing methods into a consensus classifier PredictSNP. The developed consensus of these prediction tools provided significant improvement in prediction performance over the individual tools and also over three previously developed consensus classifiers. Finally, we developed a web interface to allow an easy access to all eight prediction tools and consensus PredictSNP. Predictions from the computational tools are supplemented by experimental annotations from Protein Mutant Database [30] and UniProt database [31].

## Design and Implementation

### Prediction Tools

Initially, eight selected prediction tools were chosen for the evaluation (Table 1). The tools had to satisfy following criteria: (i) to allow submission of user-defined sequence, (ii) to be available as a stand-alone application to allow large-scale evaluations and provide stable service, and (iii) not to require a protein structure for the prediction since structural information is available only for a small portion of known sequences. Four selected tools – nsSNPAnalyzer, PhD-SNP, PPH-2, SNAP – each use a decision model trained by various machine-learning methods. Out of the remaining tools, SIFT and PANTHER use solely evolutionary information, while MAPP also considers the differences in physicochemical properties between wild-type and mutant amino acids. Finally, PPH-1 employs an expert set of empirical rules for the classification [32]. All selected tools were installed on local server and run using their default settings with the following modifications. A pipeline developed in the framework of the HotSpot Wizard server [33] was used for construction of multiple sequence alignments and a phylogenetic tree for MAPP. In short, a BLAST sequence search [34] against the non-redundant database at NCBI [35] is performed to gather protein sequences similar to the query. Sequences are clustered by CD-HIT 4.6 [36] and representatives of the clusters aligned using MUSCLE 3.8 [37]. Then, a phylogenetic tree is calculated by Rate4Site 2.01 [38]. PPH-2 offers a choice from two machine-learning models trained by Naïve Bayes on different datasets – HumDiv [13] and HumVar [13]. Only the HumDiv-trained model was employed in this study since HumDiv dataset was constructed using additional criteria to reduce the number of possibly erroneous annotations [13]. SIFT can employ two sequence databases for homologues identification: the non-redundant database at NCBI or UniProt [31], the former being used in this study. Since the SIFT algorithm is unable to process very long sequences, we automatically truncated these sequences into a fragments of 700 amino acids with analyzed mutation located in theirs centers as recommended in the user manual. Finally, median sequence conservation of SIFT method was set to three according to the recommended range.

**Table 1:** Principles and training datasets of eight evaluated tools.

Tool name	Version	Principle	Training dataset	Reference
MAPP	28.6.2005	Physicochemical properties and alignment score	No training dataset	[15]
nsSNPAnalyzer	12.2.2004	Random forest	SwissProt 3,511 neutral/502 deleterious	[11]
PANTHER	1.02	Hidden Markov model and alignment score	No training dataset	[16], [54]
PhD-SNP	2.06	Support vector machine	SwissProt 17,983 neutral/16,330 deleterious	[17]
PolyPhen-1	1.18	Expert set of empirical rules	No training dataset	[12]
PolyPhen-2	2.2.2	Naïve Bayes	SwissProt, dbSNP 7,070 neutral/5,322 deleterious	[13]
SIFT	4.0.4	Alignment score	No training dataset	[59]
SNAP	1.1.30	Neural network	SwissProt, Protein Mutant Database 40,830 neutral/39,987 deleterious	[14]

### Datasets

#### Benchmark dataset

The benchmark dataset used for the evaluation of the selected prediction tools and training of consensus classifier PredictSNP was compiled from five different sources. The first four constitute training datasets of the tools, which were not selected for evaluation since they did not meet the selection criteria defined in the previous section. These sources were following: SNPs&GO [19] dataset of 58,057 mutations compiled from SwissProt, the MutPred [10] dataset of 65,654 mutations compiled from SwissProt and HGMD [39] and the PON-P [28] training dataset of 39,670 mutations compiled from dbSNP, PhenCode [40], IDbases [41] and 16 individual locus-specific databases. Since only the HumDiv-trained model of PPH-2 was employed in this study, we could include its second dataset HumVar containing 41,918 mutations from SwissProt and dbSNP into the benchmark dataset. The final source was Humsavar [42], which is a distinct unit of UniProt containing 36,994 neutral and disease-related mutations found in UniprotKB/SwissProt entries. Such combination of sources should result into a large and diverse dataset. On the other hand, this dataset will certainly contain many duplicate records as some particular mutations are in more than one source datasets. Moreover, this dataset will definitely have large overlaps with the training datasets of prediction tools selected for evaluation. To resolve these issues and thus construct fully independent dataset, we applied following procedures. Pairs of mutations with the conflict functional annotations were purged, e.g., one particular mutation is considered as a deleterious in one source dataset, but neutral in another source dataset. All duplicate mutations were excluded. The training datasets of all evaluated prediction tools were collected and mutations overlapping between the training datasets and our dataset were removed to create fully independent PredictSNP benchmark dataset. All selected prediction tools use at least some position-specific parameters derived from evolutionary information as significant indicators of pathogenicity. Therefore, we removed both directly overlapping mutations and mutations at any overlapping positions, i.e., positions which were mutated in the training datasets of selected prediction tools. The positions were considered overlapping if they were located in the fragments of two sequences aligned by BLAST search with e-value 10^−10^ and the aligned fragments had at least 50% identity. Finally, all mutations at positions overlapping with testing datasets described in the next section were removed to assure independence between PredictSNP benchmark and the testing datasets. As a complement to the independent PredictSNP benchmark dataset, another dataset containing only mutations present also in the training sets of evaluated tools (nsSNPAnalyzer, PhD-SNP, PolyPhen-2 and SNAP) was compiled. The OVERFIT dataset was compiled to estimate the effect of the overlap between the training datasets of evaluated tools and the testing dataset on performances of these tools.

#### Testing datasets

Two testing datasets were prepared for evaluation of PredictSNP performance. The Protein Mutant Database (PMD) dataset was derived from PMD (version 07Mar26) which contains experimental information about effects of 165,880 single point mutations on protein activity, stability and connections to diseases; extracted from more than 10,000 articles [30]. The records with annotations [ = ], i.e., no change of activity, were considered as neutral, while the records with any other annotations were considered as deleterious. All mutations with conflicting annotations, e.g., [ = ] and [++] at the same time, were excluded. Similar to the process employed during the preparation of the PredictSNP benchmark dataset, all mutations at the positions overlapping with the training datasets of the evaluated tools were removed. The subset of PMD testing dataset containing only the mutations associated with sequences from the UniProt database, called PMD-UNIPROT, was prepared to enable the evaluation by CONDEL during the comparison of PredictSNP classifier to other consensus classifiers.

The second testing dataset was compiled from experimental studies summarized by Yampolsky and Stoltzfus [43]. One protein out of twelve was excluded due to very short length (30 amino acid residues). This set was complemented by mutations gathered from two patent applications issued by Danisco Inc. describing the effect of mutations on serine protease from *Bacillus subtilis* [44] and alpha-amylase from *Geobacillus stearothermophilus* [45]. This dataset of thirteen massively mutated proteins (MMP) initially contained 16,500 mutations. For all mutations originating from experimental studies, change of activity is provided in the form of a categorical value. In correspondence with the construction of PMD testing dataset, only the mutations maintaining the wild-type level of activity were considered as neutral. In the case of patent applications, the specific ratio of activity change for each mutation is known, and accordingly to the information enclosed in the source materials, the mutations with the activity changes larger than 50% were considered as deleterious. Finally, the mutations at the positions overlapping with training datasets of the evaluated tools or PMD testing dataset were removed from MMP dataset.

### Consensus Classifier

A key step for the development of the consensus classifier is the design and implementation of computational framework, which defines the way of combining the results from the individual tools. In the previous steps, we prepared the datasets and selected the tools suitable for their evaluation. Then, we retrieved predicted effects of mutations together with their corresponding confidence scores, which reflect the degree to which individual tools trust to their predictions. With the exception of PPH-1, all of the integrated tools classify the effect of mutation into two classes: neutral or deleterious. PPH-1 provides extra class of possibly deleterious, which can be considered as a deleterious class with the confidence score equal to 0.5. Unfortunately, all integrated tools use different scales for reporting their confidence scores, which renders their combination and direct comparison problematic. Therefore, we transformed the individual confidence scores to one comparable scale in range of 0–100% using the values of their observed accuracies, which express the fraction of correct predictions by a given tool on the particular level of confidence. The transformation functions defining the relationships between the confidence score of each tool and their corresponding observed accuracies were derived using PredictSNP benchmark dataset. For the integrated tools providing the confidence score in a form of categorical value (PhD-SNP, PolyPhen-1 and SNAP), the observed accuracies were calculated as the number of correct predictions to the number of all predictions separately for each category. For the remaining tools, all evaluated mutations from PredictSNP dataset were sorted by the continuous value, indicating confidence score and consequently partitioned into 60 bins consisting of equivalent number of members. Finally, these bins were averaged over five neighboring bins. Since the relationship between the confidence score and the observed accuracy can be different for deleterious and neutral prediction classes, the transformation functions were developed for both classes separately (Figure S1). After the overall predictions and corresponding transformed confidence scores were obtained, the PredictSNP consensus prediction was calculated using the following equation:
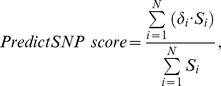
where *N* is the number of integrated tools, 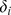 represents the overall prediction (+1 for the deleterious prediction, −1 for the neutral prediction) and 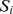 expresses the transformed confidence scores. The output value of PredictSNP score belongs to the continuous interval <−1, +1>. The mutations are considered to be neutral for the values in the interval <−1, 0> and deleterious for the values in the interval (0, +1>. The absolute distance of the PredictSNP score from zero expresses the confidence of the consensus classifier about its prediction. For easy comparison with the confidence scores of individual integrated tools, we transformed the confidence of the PredictSNP consensus classifier to the observed accuracy in the same way as described for confidence scores of the integrated tools (Figure S1).

Besides implementing the advanced weighted majority vote model, the induction of the consensus model was performed by using six different machine learning methods of WEKA 3.75 software package [46]. The first selected method was the Naïve Bayes (*weka.classifiers.bayes.NaiveBayes*), representing a probabilistic classifier based on the Bayesian theorem [47]. As a representative of the class of regression analysis models, we used the multinomial logistic regression model with a ridge estimator (*weka.classifiers.functions.Logistic*) [48]. Neural networks were represented by the voted perceptron algorithm in the implementation by Freund and Schapire (*weka.classifiers.functions.VotedPerceptron*) [49]. From the class of Support Vector Machine (SVM) classifiers, the SVM with polynomial kernel function as implemented in LIBSVM was selected (*weka.classifiers.functions.LibSVM*) [50]. The K-nearest neighbor classifier represented the class of classifiers based on the assumption that similar cases belong to the same class (*weka.classifiers.lazy.IBk*) [51]. Finally, the ensemble-based approach – Random forest – was selected, which constructs set of decision trees and the classification is based on the consensus of their decisions (*weka.classifiers.trees.RandomForest*) [52]. All models were derived using the default parameters.

## Results

### Construction of Datasets

In this study, we performed an evaluation of eight tools for prediction of the effects of mutations on protein function and combined six of them into the consensus classifier PredictSNP (for explanation of employed evaluation metrics see Supporting text S1). The proper benchmark dataset is of prime importance for the evaluation of prediction tools since overlaps between the composition of the benchmark dataset and the training datasets of a tool would result into overly optimistic performance evaluation of such tool [22], [23]. These overlaps can also hinder the construction of consensus classifier as an unwarranted degree of significance could be given to the tools with overlap between datasets [22]. For these reasons, we strived to secure the full independence of the PredictSNP benchmark dataset for unbiased evaluation of selected tools and proper training of our consensus classifier. The same care was also taken when preparing both testing datasets for the comparison of performance of PredictSNP consensus classifier, its constituent tools and other consensus classifiers.

**Figure 1:**
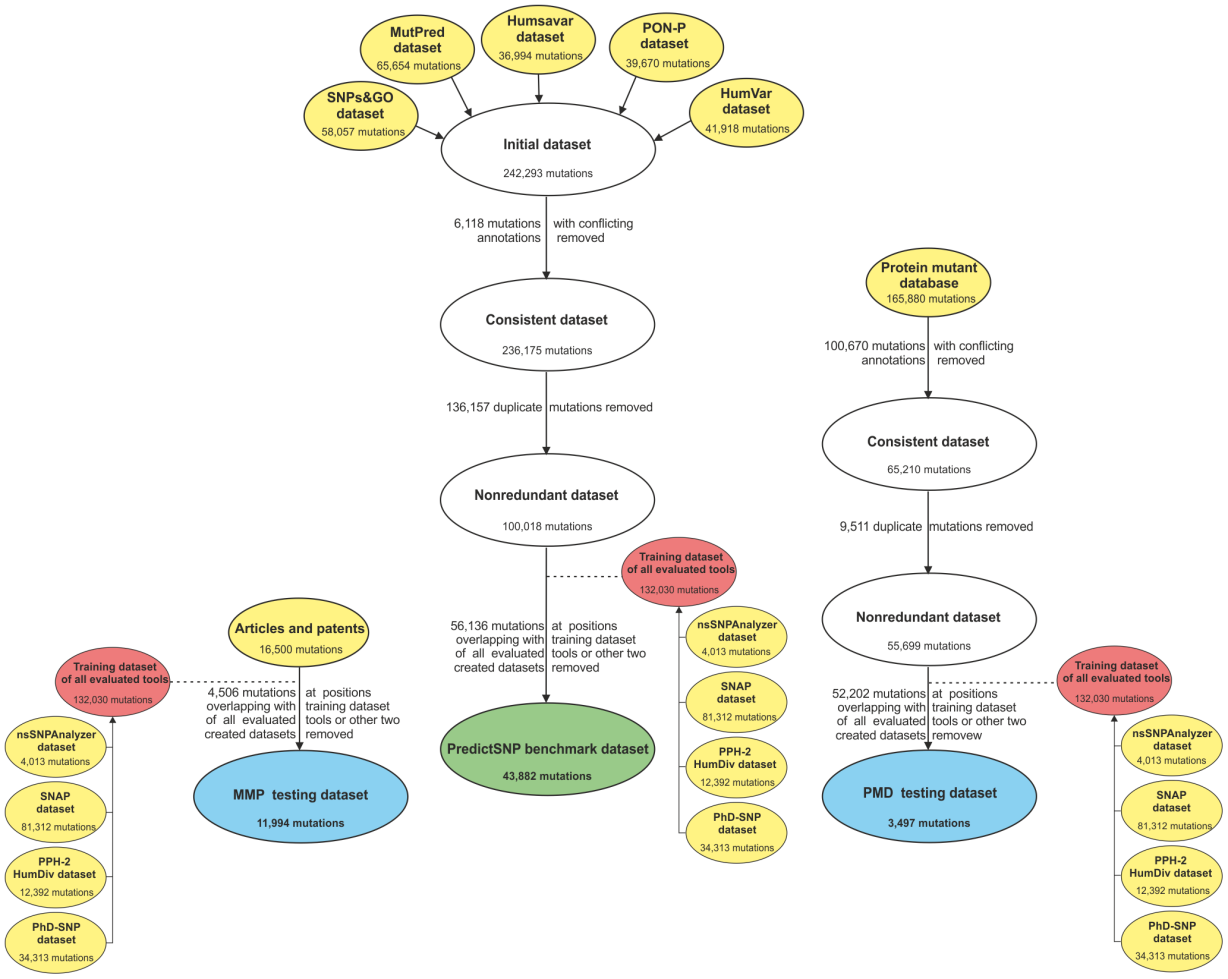
Workflow diagram describing construction of independent datasets. The various sources of mutation data are shown in yellow, intermediate datasets in white, Protein Mutant Database (PMD) testing dataset and the testing dataset compiled from studies on massively mutated proteins (MMP) in blue, and PredictSNP benchmark dataset in green. The data from the original training datasets of all evaluated tools shown in red were removed from newly constructed datasets.

The independent benchmark dataset was combined from five redundant datasets by removing all duplicates and subtracting all mutations present at the positions used in the training of the evaluated tools or in any of the two testing datasets (Figure 1). This procedure resulted in the PredictSNP benchmark dataset of 43,882 mutations (24,082 neutral and 19,800 deleterious) in the 10,085 protein sequences (Dataset S1). Complementary OVERFIT dataset was compiled from mutations present in the training sets of evaluated tools (Dataset S2). This dataset contained 32,776 mutations (15,081 neutral and 17,695 deleterious) in the 6,889 protein sequences.

Similarly, two testing datasets for evaluation of consensus classifier were prepared from Protein Mutant Database (PMD) and studies on massively mutated proteins (MMP) (Figure 1). The testing datasets consisted of 3,497 mutations (1,248 neutral and 2,249 deleterious) in 1,189 protein sequences for PMD dataset (Dataset S3) and 11,994 mutations (4,456 deleterious and 7,538neutral) in 13 protein sequences for MMP dataset (Dataset S4). The PMD-UNIPROT subset of PMD dataset with mapping on UniProt database was compiled from 1,430 mutations (518 neutral and 912 deleterious) in the 433 protein sequences.

**Figure 2:**
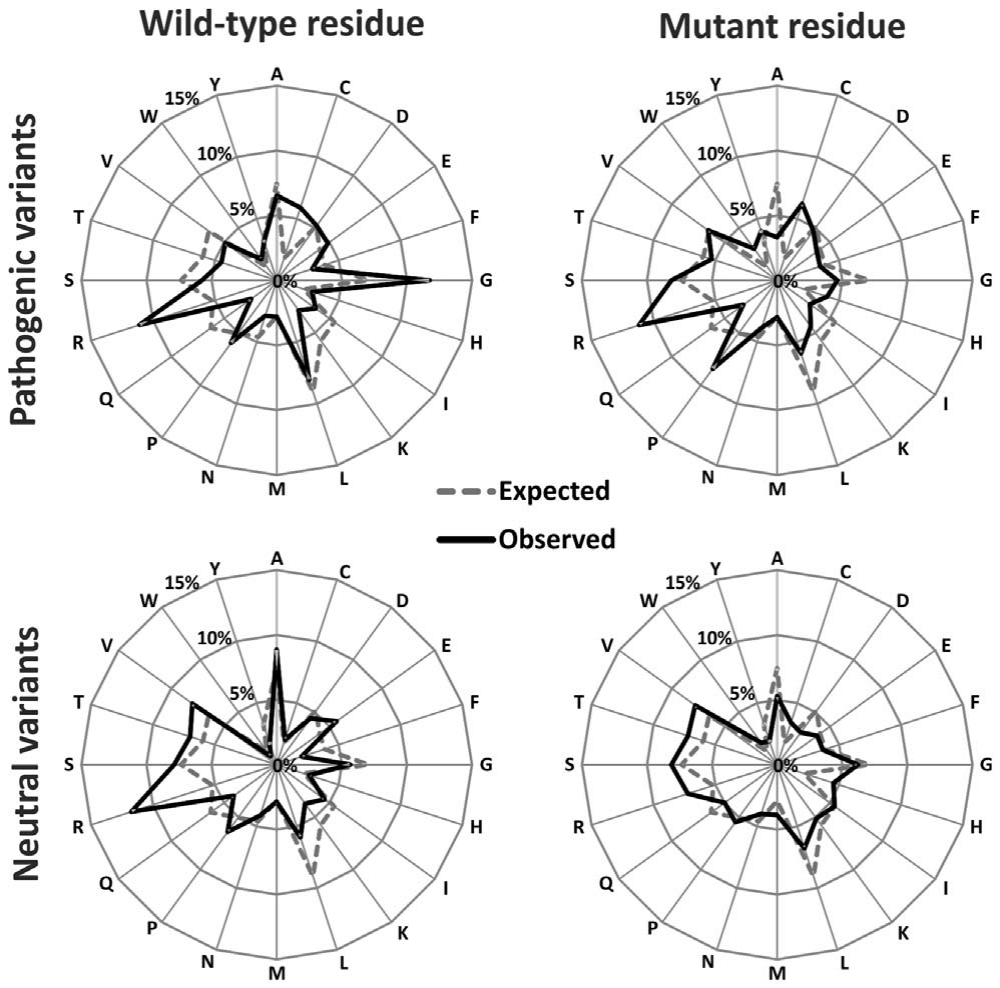
Distribution of amino acids in PredictSNP benchmark dataset. Expected distributions of amino acid residues were extracted from 105,990 sequences in the non-redundant OWL protein database (release 26.0) [58].

The distributions of wild-type and mutant residues for all four datasets were compared with the expected distributions (Table S1, S2, S3, S4) and the Pearson correlation coefficients between observed and expected distributions were calculated. This analysis showed that all datasets are biased. Following correlation coefficients were observed: 0.69 for OVERFIT dataset, 0.54 for PredictSNP benchmark dataset, 0.52 for MMP dataset and 0.21 for PMD dataset. In the case of PMD dataset, the observed bias is largely due to fivefold overrepresentation of alanine in the mutant distribution - an obvious consequence of the frequent use of alanine scanning technique. Although the weak correlation calculated for PredictSNP benchmark suggested considerable differences between observed and expected distribution, the individual deviations for particular amino acids are rarely extreme (Figure 2) with the average 33% difference from the expected numbers (Table S1). The most striking difference was observed for arginine and cysteine, which were twice more frequently present in the wild-type distribution, while cysteine and tryptophan were twice more frequently present in the mutant distribution (Table S1). Underrepresentation by more than 25% was observed for phenylalanine, lysine and glutamine in the wild-type distribution and alanine, glutamine, leucine and aspartic acid in the mutant distribution (Table S1).

### Evaluation of Individual Prediction Tools

The performance of individual prediction methods was compared using the PredictSNP benchmark dataset (Table 2 and S5). The evaluation showed that the applicability of some of the tools is limited to only a part of the dataset. 66% of the dataset was not evaluated by nsSNPAnalyzer due to a requirement for the existence of a homologous protein to the investigated sequence in the ASTRAL database [53], a condition which was not fulfilled by many protein sequences in PredictSNP benchmark dataset. PANTHER was not able to evaluate 45% of the dataset mainly due to the fact that the investigated mutations could not be found at given positions in the pre-computed multiple sequence alignments of PANTHER library [54]. In the case of MAPP, 12% of the PredictSNP benchmark dataset was not evaluated due to mutations located within gaps of multiple sequence alignments.

**Table 2:** Performance of individual and PredictSNP prediction tools with three independent datasets.

Performance metrics^a^	Dataset	MAPP	nsSNPAnalyzer	PANTHER	PhD-SNP	PPH-1	PPH-2	SIFT	SNAP	PredictSNP
Percent of evaluated mutations	PredictSNP	87.8	33.5	54.6	100.0	98.8	100.0	97.1	99.1	100.0
	PMD	81.1	63.4	38.1	100.0	97.1	98.3	77.6	95.1	100.0
	MMP	99.8	91.5	61.9	100.0	97.7	97.7	95.4	100.0	100.0
	**Overall**	**89.9**	**47.0**	**55.1**	**100.0**	**98.5**	**99.4**	**95.6**	**99.0**	**100.0**
Accuracy^b^	PredictSNP	0.711	0.632	0.642	0.746	0.682	0.701	0.723	0.670	0.747
	PMD	0.653	0.629	0.651	0.633	0.654	0.632	0.643	0.631	0.642
	MMP	0.707	0.618	0.603	0.629	0.684	0.677	0.646	0.709	0.708
	**Overall**	**0.707**	**0.629**	**0.635**	**0.715**	**0.681**	**0.692**	**0.703**	**0.676**	**0.733**
Matthews correlation coefficient^b^	PredictSNP	0.423	0.219	0.296	0.494	0.364	0.407	0.447	0.346	0.492
	PMD	0.327	0.243	0.303	0.258	0.299	0.289	0.312	0.253	0.281
	MMP	0.400	0.228	0.227	0.255	0.357	0.359	0.308	0.406	0.408
	**Overall**	**0.413**	**0.223**	**0.282**	**0.432**	**0.358**	**0.390**	**0.411**	**0.353**	**0.463**
Area under the receiver operating characteristics curve^b^	PredictSNP	0.773	0.634	0.692	0.812	0.695	0.776	0.784	0.732	0.808
	PMD	0.695	0.630	0.697	0.676	0.658	0.704	0.685	0.667	0.700
	MMP	0.759	0.620	0.676	0.685	0.720	0.774	0.710	0.769	0.787
	**Overall**	**0.766**	**0.631**	**0.689**	**0.778**	**0.698**	**0.771**	**0.763**	**0.735**	**0.797**

PPH-1 – PolyPhen-1; PPH-2 – PolyPhen-2; PMD dataset – dataset from Protein Mutant Database; MMP – dataset of massively mutated proteins;

^a^– detailed evaluation is available in Tables S5, S6, S7;

b – these metrics were calculated with normalized numbers.

Concerning the overall performance of individual tools, PANTHER and nsSNPAnalyzer exhibited significantly lower accuracies, Matthews correlation coefficients and area under the receiver operating characteristics curve (AUC) than other evaluated tools on PredictSNP benchmark dataset (Table 2). The other six evaluated prediction tools achieved very good performances with the accuracy ranging from 0.68 to 0.75, and Matthews correlation coefficient ranging from 0.35 to 0.49.

Additionally, we assessed the effect of the dataset independence on the tool performance. The individual tools were evaluated with OVERFIT dataset containing only the mutations from the training datasets of the evaluated tools (Table S8). In comparison with the independent dataset, the increase of accuracy by 5% was observed for PPH-2 and SNAP. The most striking difference was measured for PhD-SNP for which the accuracy increased by more than 11%. Training dataset of PhD-SNP constituted over 94% of the OVERFIT dataset.

The performances of individual tools observed with PredictSNP benchmark dataset were in good correspondence with a recent comprehensive evaluation of nine prediction methods by Thusberg *et al.* [25]. The differences in performance can be attributed to differences in benchmark datasets, and the fact that a fully independent dataset has not been used for evaluation due to the inaccessibility of training datasets for several evaluated tools [25]. We analyzed benchmark dataset of Thusberg *et al.* in detail and found out that only about 33% of mutations (13,467 cases) is shared with our benchmark dataset and about 56% of mutations (22,652 cases) is shared with training sets of evaluated tools, i.e., MutPred, nsSNPAnalyzer, PhD-SNP, PolyPhen-2 and SNAP, SNPs&GO (Table S9). Despite of these differences in composition of the benchmark datasets, all shared tools differ in accuracy by less than 5% with the exception of SIFT and PANTHER. The difference of about 7% for SIFT can be explained by different settings or selection of different database for identification of homologues. In the case of PANTHER, the difference of about 12% can be caused by newer version of the decision core (cSNP 1.02 instead of cSNP 1.00) and updated version of PANTHER library (7.2 instead of 6.0).

### Development of Consensus Classifier

**Figure 3:**
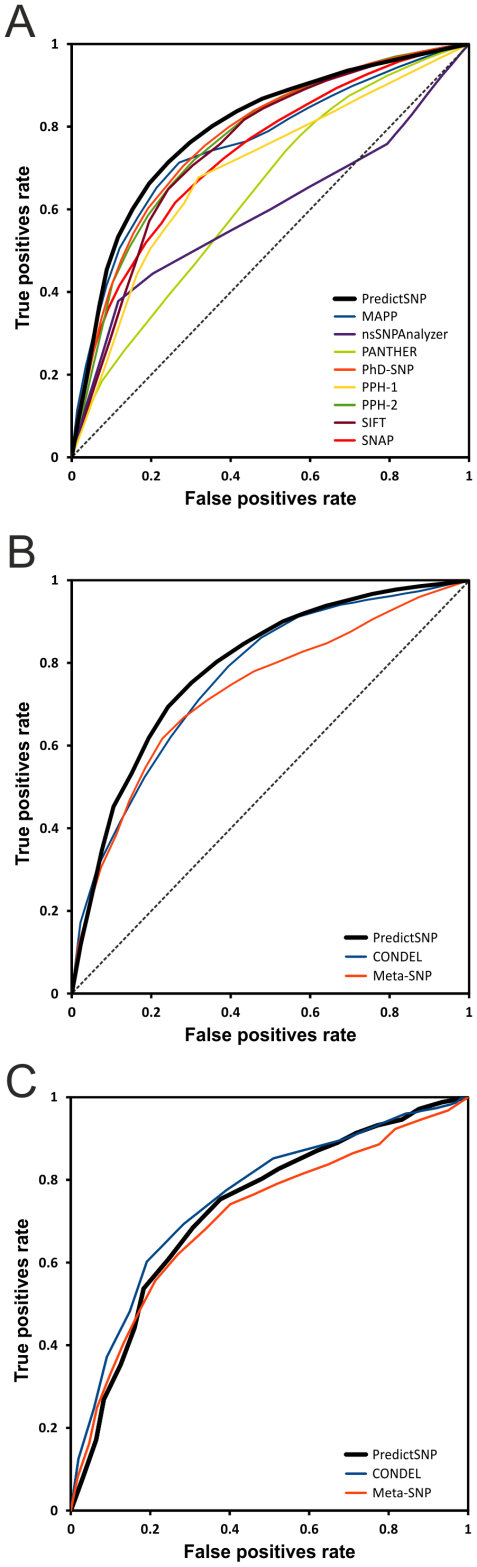
Overall receiver operating characteristic curves for all three independent datasets. Comparison of PredictSNP and its constituent tools with PredictSNP benchmark dataset (A). Comparison of PredictSNP and other consensus classifiers with MMP data set (B) and PMD-UNIPROT dataset (C). The dashed line represents random ranking with AUC equal to 0.5.

With the exception of PANTHER and nsSNPAnalyzer, all other six evaluated tools were selected for the development of consensus classifier. The prediction tools employed in the consensus system should be as accurate as possible and also have different decision boundaries. Therefore, we verified the absence of strong correlation between any pair of tools that could negatively affect the consensus prediction (Table S10). To identify the most suitable method for combining the selected tools, we trained seven consensus classifiers on the PredictSNP benchmark dataset using seven machine learning methods, which represent the most important classification principles [55]. To our surprise, none of the methods provided a clearly superior performance despite very different level of complexity of employed model (Table S11). The majority vote weighted by the transformed confidence scores of the integrated tools provided the most balanced performance over the investigated datasets. Therefore, motivated by its good performance and small probability of over-fitting [56], [57], we utilized the majority vote weighted by the transformed confidence scores for the development of our consensus classifier PredictSNP. The comparison of overall performance of the PredictSNP classifier and its integrated tools over all three independent datasets showed that the combining these tools into the consensus lead to significantly improved prediction with respect to the best of the integrated tools (Table 2 and Figure 3A). Since a single tool could be the best choice for one dataset and moderate or even a poor choice for another dataset (Table 2), the combination of their predictions by PredictSNP represents a robust alternative for users who are not experts on the prediction tools or miss information about involvement of studied protein in the training of some particular tool.

### Comparison of PredictSNP with Other Consensus Classifiers

The performance of newly developed PredictSNP was compared to other consensus classifiers CONDEL, PON-P and Meta-SNP using the PMD-UNIPROT and MMP testing datasets. The nature of these datasets is very different. While PMD-UNIPROT dataset contains a large number of proteins with only about three mutations per protein, MMP dataset consists of only a few proteins associated with a very large number of mutations. CONDEL, Meta-SNP and PredictSNP consensus tools were able to evaluate almost all mutations from both PMD-UNIPROT and MMP testing datasets enabling their mutual comparison (Table 3, Table S12 and Figure 3B,C). The best performing tool on PMD-UNIPROT testing dataset varies according to the evaluation metrics. The highest accuracy (0.679) and Matthews coefficient (0.366) was observed for PredictSNP, while CONDEL achieved better result for AUC. For MMP dataset, the results confirmed a significantly improved performance of PredictSNP in all three employed metrics. Obtained significant difference in the accuracy of Meta-SNP and CONDEL is in good correspondence with 4% difference previously reported for the comparison of these tools with the NSV-2012 dataset consisting of 972 mutations from the SwissVar database [29].

**Table 3:** Performance of consensus classifiers with PMD-UNIPROT and MMP datasets.

Performance metrics^a^		PMD-UNIPROT			MMP		
		CONDEL	Meta-SNP	PredictSNP	CONDEL	Meta-SNP	PredictSNP
Percent of evaluated mutations		100.0	100.0	100.0	100.0	99.7	100.0
Accuracy^b^		0.562	0.670	0.679	0.640	0.673	0.708
Matthews correlation coefficient^b^		0.202	0.343	0.366	0.349	0.351	0.433
Area under the receiver operating characteristics curve^b^		0.755	0.709	0.732	0.770	0.730	0.780

a – detailed evaluation is available in Table S12;

b – these metrics were calculated with normalized numbers.

The prediction by PON-P was obtained only for 62% and 58% of PMD-UNIPROT and MMP datasets, respectively. This is because PON-P assigns the effect of mutations as unknown for cases with less reliable prediction. To evaluate the benefit of PON-P approach, we compared the performance of PON-P with modified version of PredictSNP, which returned predictions for the same number of mutations as PON-P (Table S13). These mutations have the highest PredictSNP score reflecting the degree of confidence in its own decision. In the case of PMD-UNIPROT dataset, the accuracy of modified PredictSNP predictions was increased by 3.7% to 71.6%, compared to PON-P accuracy 72.9%. In the case of MMP dataset, the accuracy of modified PredictSNP was increased by 7.6% to 78.4%, compared to PON-P accuracy 75.7%. The significant reduction in the number of evaluated mutations led to the large improvement in the prediction accuracy.

### Description of Web Server

**Figure 4:**
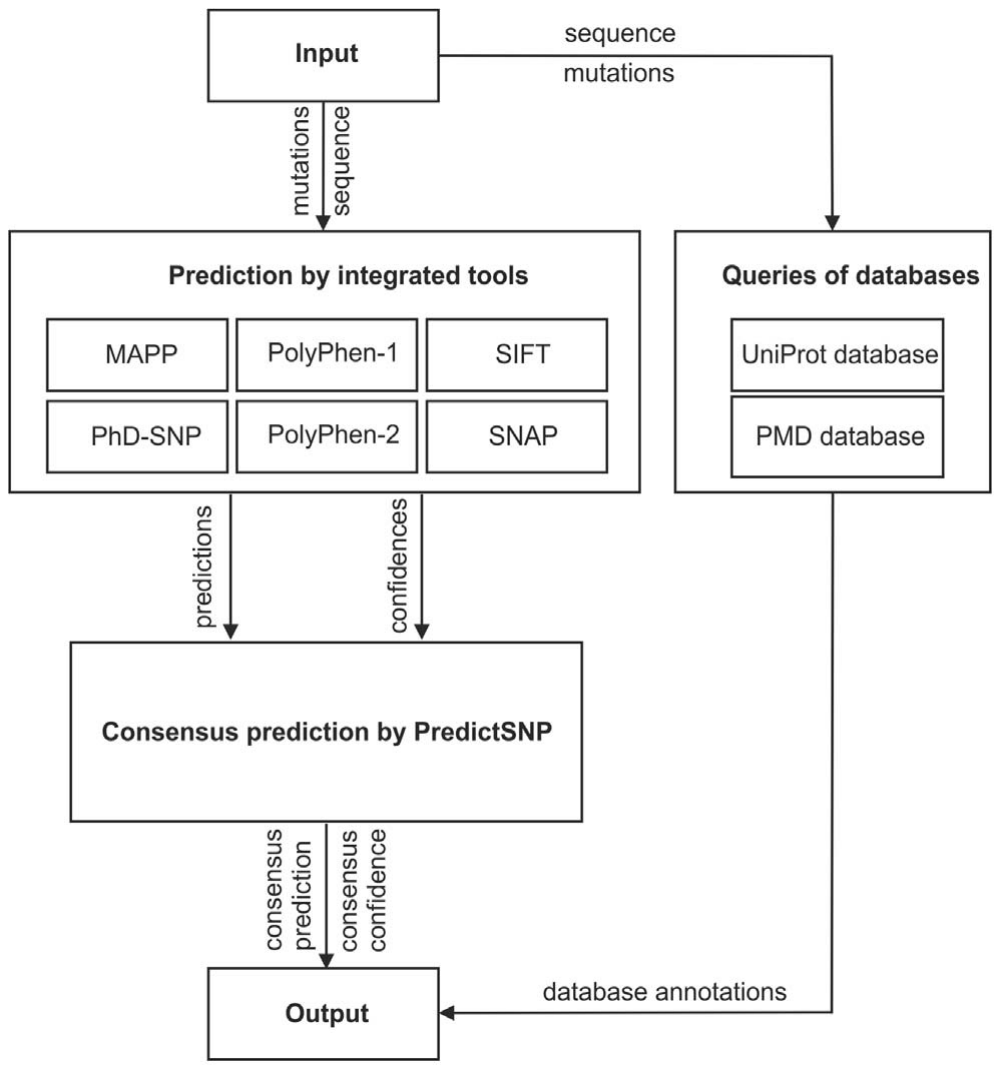
Workflow diagram of PredictSNP. Upon submission of the input sequence and specification of investigated mutations, integrated predictors of pathogenicity are employed for evaluation of the mutation and the consensus prediction is calculated. In the meantime, UniProt and PMD databases are queried to gather the relevant annotations.

Web interfaces are currently available for six of the tools evaluated in our study. However, some of the interfaces allow the input of only a single mutation. Moreover, MAPP and PPH-1 have to be installed and run locally since there are currently no web interfaces available for these tools. To facilitate the access of users to the predictions from all eight individual tools and the robust consensus classifier PredictSNP, we developed a web interface to allow comfortable submission of jobs and retrieval of the results from the individual tools and databases as well as the consensus classifier PredictSNP (Figure 4).

**Figure 5:**
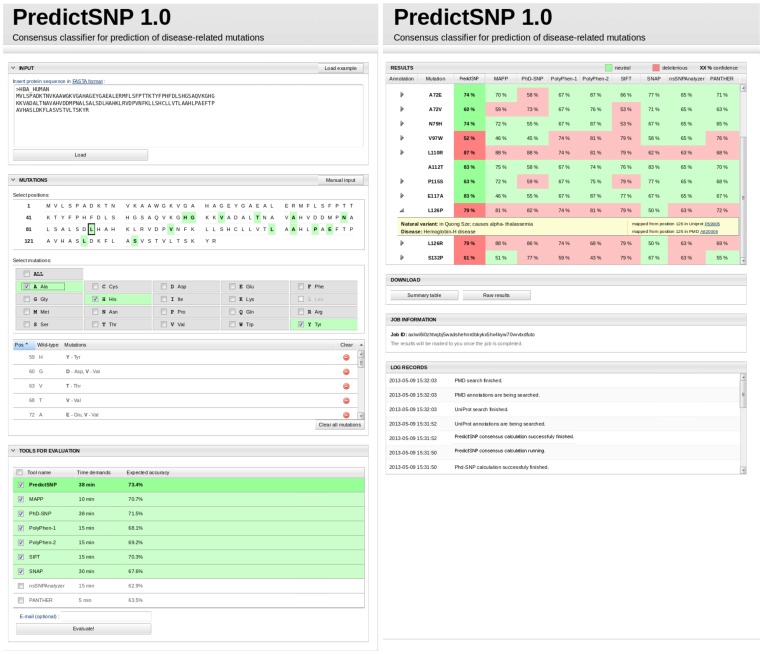
Graphic user interface of PredictSNP. The web server input (left) and output (right) page.

Using this web server, a user can load an amino acid sequence of a query protein in FASTA format, select positions for mutations and desired mutations using the input page (Figure 5). Alternatively, the user can submit a list of mutations in a text format. After all desired mutations are specified, the user can select tools to be employed for the evaluation of selected mutations. A time estimate is provided for each tool and a number of mutations, based on an average evaluation time for individual tools.

The server then runs the prediction using all selected tools. In the cases where MAPP is included in the selection, the necessary multiple sequence alignment and phylogenetic tree are automatically calculated. The confidence scores of integrated tools are transformed to observed accuracies and together with corresponding prediction combined into PredictSNP prediction using the weighted majority vote consensus. The prediction is finalized by calculation of the PredictSNP confidence score. To provide a full picture about an inferred effect of mutation on protein function, the predictions are complemented with the experimental annotations. The UniProt and the Protein Mutant Databases are queried for any annotation regarding mutated positions in closely homologous proteins with identity over 95%, supplying information on the importance of the given position for the protein function, the overview of known natural variations at the position, the experimentally characterized mutations at the position as well as their connection to disease.

The predictions of individual tools and the consensus prediction by PredictSNP for all selected mutations are provided together with the confidence of these predictions on the output page (Figure 5). If available, the experimental annotations for studied mutations are provided along with links to respective records in the databases to complement the prediction. The user can download the summary of results in the form of a comma separated values (CSV) file or the detailed results including also the output files from individual tools as a single zip file.

## Availability and Future Directions

The PredictSNP web server is freely available to the community at http://loschmidt.chemi.muni.cz/predictsnp. The developed datasets (Dataset S1, S2, S3, S4), the user manual (Supporting text S2) and standalone version of PredictSNP consensus calculator (Software S1) are also available from the website. The standalone version represents an alternative to web server that is suitable for massive mutagenesis studies. In contrast to the online version, the standalone version requires pre-calculated predictions from all six integrated tools as an input. For the best performance, a user should use the same version and settings of integrated tools as described in the method section.

Concerning the future development, authors plan to assess new emerging tools for the prediction of the effect of mutations and to consider integrating any stand-alone tool that would provide additional improvement in the collective prediction. Particular attention will be focused on the tools employing principles or attributes not considered by currently integrated tools, e.g. mutations on the correlated positions, protein-protein interaction sites and others.

## Supporting Information

Dataset S1 Composition of PredictSNP benchmark dataset.
(XLSX)

Dataset S2 Composition of OVERFIT testing dataset.
(XLSX)

Dataset S3 Composition of PMD testing dataset.
(XLSX)

Dataset S4 Composition of MMP testing dataset.
(XLSX)

Figure S1 Transformation functions between confidence score of individual tools and developed consensus classifier, and observed accuracies of these tools on PredictSNP benchmark dataset.
(TIF)

Software S1 Standalone version of PredictSNP for calculation of consensus prediction.
(GZ) Click here for additional data file.

Table S1 Composition of PredictSNP benchmark dataset.
(PDF)

Table S2 Composition of PMD testing dataset.
(PDF)

Table S3 Composition of MMP testing dataset.
(PDF)

Table S4 Composition of OVERFIT testing dataset.
(PDF)

Table S5 Performance of prediction tools with PredictSNP benchmark dataset.
(PDF)

Table S6 Performance of prediction tools with PMD testing dataset.
(PDF)

Table S7 Performance of prediction tools with MMP testing dataset.
(PDF)

Table S8 Performance of prediction tools with OVERFIT testing dataset.
(PDF)

Table S9 Comparison of performance evaluation with Thusberg dataset and PredictSNP benchmark dataset.
(PDF)

Table S10 Pairwise correlation of integrated tools.
(PDF)

Table S11 Performance of selected machine learning methods with PredictSNP, PMD and MMP datasets.
(PDF)

Table S12 Performance of consensus classifiers with PMD-UNIPROT and MMP datasets.
(PDF)

Table S13 Performance of consensus classifiers PON-P and PredictSNP with PMD-UNIPROT and MMP datasets.
(PDF)

Text S1 Performance evaluation metrics.
(PDF)

Text S2 PredictSNP user guide.
(PDF)
