# Supplementary figures and images for: PredictSNP: Robust and Accurate Consensus Classifier for Prediction of Disease-Related Mutations
Source: PLoS Comput Biol. 2014 Jan 16;10(1):e1003440. doi: 10.1371/journal.pcbi.1003440 (PMC3894168; doi:10.1371/journal.pcbi.1003440)

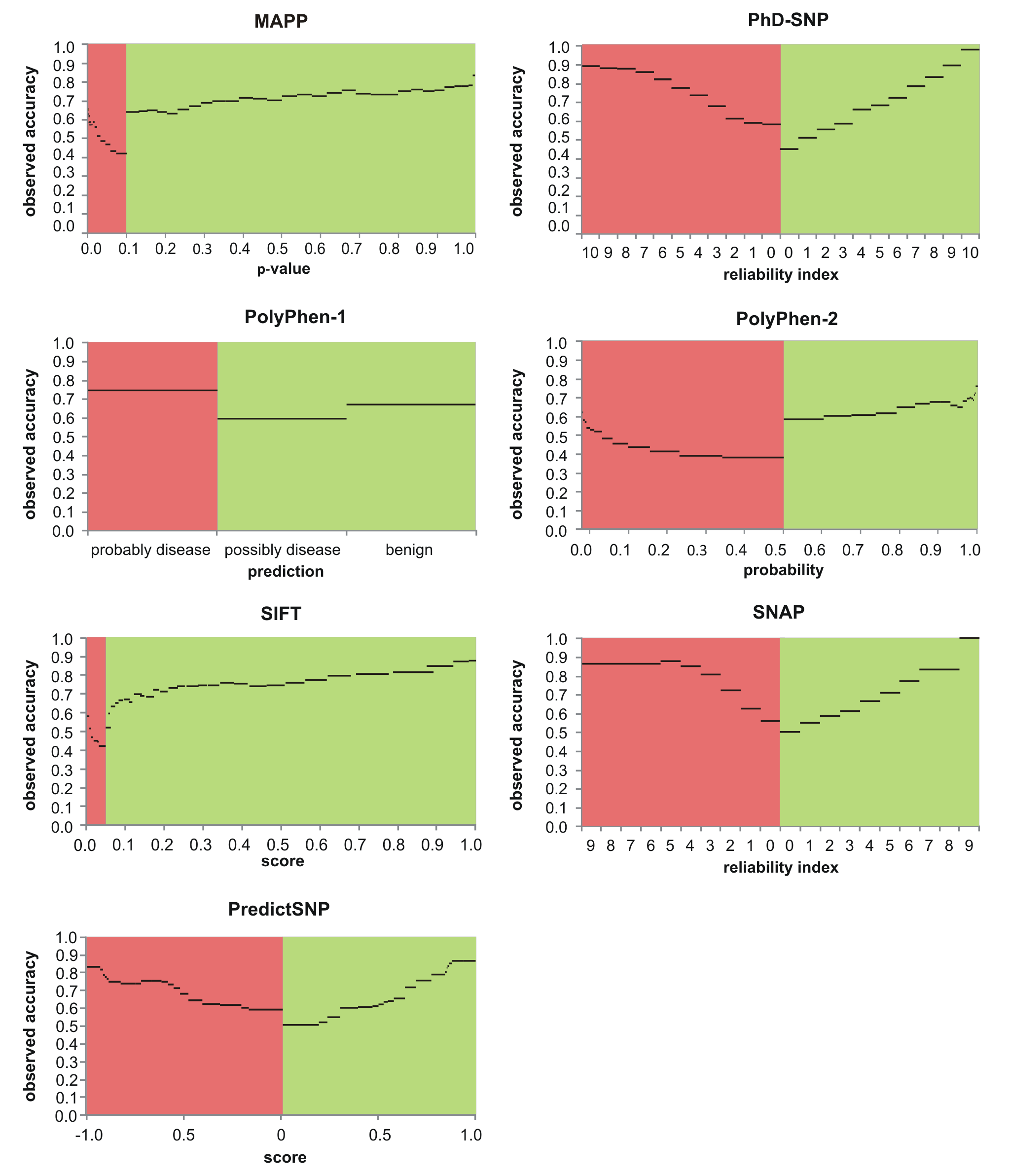

Supplement: Figure S1 — Transformation functions between confidence score of individual tools and developed consensus classifier, and observed accuracies of these tools on PredictSNP benchmark dataset. (TIF) [file pcbi.1003440.s005.tif]
