# Supplementary material for: PredictSNP: Robust and Accurate Consensus Classifier for Prediction of Disease-Related Mutations
Source: PLoS Comput Biol. 2014 Jan 16;10(1):e1003440. doi: 10.1371/journal.pcbi.1003440 (PMC3894168; doi:10.1371/journal.pcbi.1003440)
Supplement: Table S1 — Composition of PredictSNP benchmark dataset. (PDF) [file pcbi.1003440.s007.pdf]

Table S1. Composition of PredictSNP benchmark dataset.

Amino acid residues	Wild-type								
	Pathogenic variants			Neutral variants			All variants		
	Observed	Expected [a]	O/E ratio [b]	Observed	Expected [a]	O/E ratio [b]	Observed	Expected [a]	O/E ratio [b]
Ala	1,296	1,475	88%	2,134	1,794	119%	3,430	3,268	105%
Arg	2,196	1,022	215%	2,826	1,244	227%	5,022	2,266	222%
Asn	570	904	63%	994	1,100	90%	1,564	2,005	78%
Asp	1,032	1,022	101%	1,064	1,244	86%	2,096	2,266	92%
Cys	1,165	354	329%	489	430	114%	1,654	784	211%
Gln	480	1,239	39%	981	1,507	65%	1,461	2,745	53%
Glu	962	806	119%	1,369	980	140%	2,331	1,787	130%
Gly	2,326	1,396	167%	1,363	1,698	80%	3,689	3,094	119%
His	567	433	131%	632	526	120%	1,199	959	125%
Ile	731	1,081	68%	1,094	1,315	83%	1,825	2,397	76%
Leu	1,598	1,789	89%	1,422	2,176	65%	3,020	3,966	76%
Lys	566	1,140	50%	896	1,387	65%	1,462	2,527	58%
Met	553	551	100%	686	670	102%	1,239	1,220	102%
Phe	567	767	74%	483	933	52%	1,050	1,700	62%
Pro	1,183	1,003	118%	1,541	1,220	126%	2,724	2,222	123%
Ser	1,137	1,455	78%	1,896	1,770	107%	3,033	3,225	94%
Thr	885	1,180	75%	1,681	1,435	117%	2,566	2,615	98%
Trp	395	256	154%	189	311	61%	584	567	103%
Tyr	624	649	96%	415	789	53%	1,039	1,438	72%
Val	967	1,278	76%	1,927	1,554	124%	2,894	2,833	102%
All	19,800	19,800		24,082	24,082		43,882	43,882	
**Amino acid residues**	**Mutant**								
	**Pathogenic variants**			**Neutral variants**			**All variants**		
	**Observed**	**Expected [a]**	**O/E ratio [b]**	**Observed**	**Expected [a]**	**O/E ratio [b]**	**Observed**	**Expected [a]**	**O/E ratio [b]**
Ala	658	1,475	45%	1,275	1,794	71%	1,933	3,268	59%
Arg	2,210	1,022	216%	1,739	1,244	140%	3,949	2,266	174%
Asn	761	904	84%	956	1,100	87%	1,717	2,005	86%
Asp	936	1,022	92%	738	1,244	59%	1,674	2,266	74%
Cys	1,230	354	347%	825	430	192%	2,055	784	262%
Gln	635	1,239	51%	1,201	1,507	80%	1,836	2,745	67%
Glu	745	806	92%	915	980	93%	1,660	1,787	93%
Gly	916	1,396	66%	1,494	1,698	88%	2,410	3,094	78%
His	801	433	185%	1,094	526	208%	1,895	959	198%
Ile	617	1,081	57%	1,312	1,315	100%	1,929	2,397	80%
Leu	1,173	1,789	66%	1,630	2,176	75%	2,803	3,966	71%
Lys	871	1,140	76%	1,234	1,387	89%	2,105	2,527	83%
Met	562	551	102%	932	670	139%	1,494	1,220	122%
Phe	686	767	89%	888	933	95%	1,574	1,700	93%
Pro	1,667	1,003	166%	1,316	1,220	108%	2,983	2,222	134%
Ser	1,607	1,455	110%	1,958	1,770	111%	3,565	3,225	111%
Thr	1,047	1,180	89%	1,736	1,435	121%	2,783	2,615	106%
Trp	594	256	232%	505	311	162%	1,099	567	194%
Tyr	786	649	121%	460	789	58%	1,246	1,438	87%
Val	1,298	1,278	102%	1,874	1,554	121%	3,172	2,833	112%
All	19,800	19,800		24,082	24,082		43,882	43,882	

[a] – Expected numbers of amino acid residues were extracted from 105,990 sequences in the non-redundant OWL protein database (release 26.0)

[b] – O/E ratio – observed to expected ratio
