# Supplementary material for: PredictSNP: Robust and Accurate Consensus Classifier for Prediction of Disease-Related Mutations
Source: PLoS Comput Biol. 2014 Jan 16;10(1):e1003440. doi: 10.1371/journal.pcbi.1003440 (PMC3894168; doi:10.1371/journal.pcbi.1003440)
Supplement: Table S2 — Composition of PMD testing dataset. (PDF) [file pcbi.1003440.s008.pdf]

**Table S2.** Composition of PMD testing dataset.

| Amino | Wild-type | | | | | | | | |
|---|---|---|---|---|---|---|---|---|---|
| acid | Pathogenic variants | | | Neutral variants | | | All variants | | |
| residues | Observed | Expected [a] | O/E ratio [b] | Observed | Expected [a] | O/E ratio [b] | Observed | Expected [a] | O/E ratio [b] |
| Ala | 45 | 168 | 27% | 42 | 93 | 45% | 87 | 260 | 33% |
| Arg | 198 | 116 | 171% | 109 | 64 | 170% | 307 | 181 | 170% |
| Asn | 59 | 103 | 57% | 72 | 57 | 126% | 131 | 160 | 82% |
| Asp | 199 | 116 | 172% | 81 | 64 | 127% | 280 | 181 | 155% |
| Cys | 137 | 40 | 343% | 72 | 22 | 327% | 209 | 63 | 334% |
| Gln | 59 | 141 | 42% | 40 | 78 | 51% | 99 | 219 | 45% |
| Glu | 113 | 92 | 123% | 116 | 51 | 227% | 229 | 142 | 161% |
| Gly | 99 | 159 | 62% | 24 | 88 | 27% | 123 | 247 | 50% |
| His | 131 | 49 | 267% | 63 | 27 | 233% | 194 | 76 | 254% |
| Ile | 94 | 123 | 76% | 38 | 68 | 56% | 132 | 191 | 69% |
| Leu | 137 | 203 | 67% | 53 | 113 | 47% | 190 | 316 | 60% |
| Lys | 203 | 130 | 156% | 109 | 72 | 151% | 312 | 201 | 155% |
| Met | 23 | 63 | 37% | 26 | 35 | 74% | 49 | 97 | 50% |
| Phe | 86 | 87 | 99% | 34 | 48 | 71% | 120 | 135 | 89% |
| Pro | 69 | 114 | 61% | 38 | 63 | 60% | 107 | 177 | 60% |
| Ser | 143 | 165 | 87% | 124 | 92 | 135% | 267 | 257 | 104% |
| Thr | 96 | 134 | 72% | 60 | 74 | 81% | 156 | 208 | 75% |
| Trp | 90 | 29 | 310% | 23 | 16 | 144% | 113 | 45 | 250% |
| Tyr | 188 | 74 | 254% | 77 | 41 | 188% | 265 | 115 | 231% |
| Val | 80 | 145 | 55% | 47 | 81 | 58% | 127 | 226 | 56% |
| All | 2,249 | 2,249 | | 1,248 | 1,248 | | 3,497 | 3,497 | |

| Amino | Mutant | | | | | | | | |
|---|---|---|---|---|---|---|---|---|---|
| acid | Pathogenic variants | | | Neutral variants | | | All variants | | |
| residues | Observed | Expected [a] | O/E ratio [b] | Observed | Expected [a] | O/E ratio [b] | Observed | Expected [a] | O/E ratio [b] |
| Ala | 719 | 168 | 428% | 575 | 93 | 618% | 1,294 | 260 | 497% |
| Arg | 138 | 116 | 119% | 78 | 64 | 122% | 216 | 181 | 120% |
| Asn | 125 | 103 | 121% | 29 | 57 | 51% | 154 | 160 | 96% |
| Asp | 66 | 116 | 57% | 34 | 64 | 53% | 100 | 181 | 55% |
| Cys | 48 | 40 | 120% | 21 | 22 | 95% | 69 | 63 | 110% |
| Gln | 86 | 141 | 61% | 49 | 78 | 63% | 135 | 219 | 62% |
| Glu | 109 | 92 | 118% | 42 | 51 | 82% | 151 | 142 | 106% |
| Gly | 74 | 159 | 47% | 42 | 88 | 48% | 116 | 247 | 47% |
| His | 50 | 49 | 102% | 11 | 27 | 41% | 61 | 76 | 80% |
| Ile | 51 | 123 | 41% | 31 | 68 | 46% | 82 | 191 | 43% |
| Leu | 103 | 203 | 51% | 39 | 113 | 35% | 142 | 316 | 45% |
| Lys | 97 | 130 | 75% | 45 | 72 | 63% | 142 | 201 | 71% |
| Met | 31 | 63 | 49% | 19 | 35 | 54% | 50 | 97 | 51% |
| Phe | 128 | 87 | 147% | 61 | 48 | 127% | 189 | 135 | 140% |
| Pro | 49 | 114 | 43% | 12 | 63 | 19% | 61 | 177 | 34% |
| Ser | 164 | 165 | 99% | 73 | 92 | 79% | 237 | 257 | 92% |
| Thr | 65 | 134 | 49% | 26 | 74 | 35% | 91 | 208 | 44% |
| Trp | 20 | 29 | 69% | 12 | 16 | 75% | 32 | 45 | 71% |
| Tyr | 45 | 74 | 61% | 18 | 41 | 44% | 63 | 115 | 55% |
| Val | 81 | 145 | 56% | 31 | 81 | 38% | 112 | 226 | 50% |
| All | 2,249 | 2,249 | | 1,248 | 1,248 | | 3,497 | 3,497 | |

[a] – Expected numbers of amino acid residues were extracted from 105,990 sequences in the non-redundant OWL protein database (release 26.0)

[b] – O/E ratio – observed to expected ratio
