# Supplementary material for: PredictSNP: Robust and Accurate Consensus Classifier for Prediction of Disease-Related Mutations
Source: PLoS Comput Biol. 2014 Jan 16;10(1):e1003440. doi: 10.1371/journal.pcbi.1003440 (PMC3894168; doi:10.1371/journal.pcbi.1003440)
Supplement: Table S3 — Composition of MMP testing dataset. (PDF) [file pcbi.1003440.s009.pdf]

**Table S3.** Composition of MMP testing dataset.

| Amino acid residues | Wild-type | | | | | | | | |
|---|---|---|---|---|---|---|---|---|---|
| | Pathogenic variants | | | Neutral variants | | | All variants | | |
| | Observed | Expected [a] | O/E ratio [b] | Observed | Expected [a] | O/E ratio [b] | Observed | Expected [a] | O/E ratio [b] |
| Ala | 468 | 332 | 141% | 667 | 561 | 119% | 1,135 | 893 | 127% |
| Arg | 209 | 230 | 91% | 368 | 389 | 95% | 577 | 619 | 93% |
| Asn | 212 | 204 | 104% | 582 | 344 | 169% | 794 | 548 | 145% |
| Asp | 238 | 230 | 103% | 387 | 389 | 99% | 625 | 619 | 101% |
| Cys | 10 | 80 | 13% | 33 | 135 | 24% | 43 | 214 | 20% |
| Gln | 74 | 278 | 27% | 285 | 472 | 60% | 359 | 750 | 48% |
| Glu | 70 | 181 | 39% | 265 | 307 | 86% | 335 | 488 | 69% |
| Gly | 599 | 314 | 191% | 506 | 531 | 95% | 1,105 | 846 | 131% |
| His | 149 | 97 | 154% | 191 | 165 | 116% | 340 | 262 | 130% |
| Ile | 303 | 243 | 125% | 308 | 412 | 75% | 611 | 655 | 93% |
| Leu | 453 | 403 | 112% | 505 | 681 | 74% | 958 | 1,084 | 88% |
| Lys | 110 | 257 | 43% | 614 | 434 | 141% | 724 | 691 | 105% |
| Met | 96 | 124 | 77% | 130 | 210 | 62% | 226 | 333 | 68% |
| Phe | 128 | 173 | 74% | 69 | 292 | 24% | 197 | 465 | 42% |
| Pro | 196 | 226 | 87% | 349 | 382 | 91% | 545 | 607 | 90% |
| Ser | 254 | 327 | 78% | 754 | 554 | 136% | 1,008 | 881 | 114% |
| Thr | 310 | 266 | 117% | 610 | 449 | 136% | 920 | 715 | 129% |
| Trp | 47 | 58 | 81% | 93 | 97 | 96% | 140 | 155 | 90% |
| Tyr | 149 | 146 | 102% | 345 | 247 | 140% | 494 | 393 | 126% |
| Val | 381 | 287 | 133% | 477 | 487 | 98% | 858 | 774 | 111% |
| All | 4,456 | 4,456 | | 7,538 | 7,538 | | 11,994 | 11,994 | |

| Amino acid residues | Mutant | | | | | | | | |
|---|---|---|---|---|---|---|---|---|---|
| | Pathogenic variants | | | Neutral variants | | | All variants | | |
| | Observed | Expected [a] | O/E ratio [b] | Observed | Expected [a] | O/E ratio [b] | Observed | Expected [a] | O/E ratio [b] |
| Ala | 235 | 332 | 71% | 591 | 561 | 105% | 826 | 893 | 92% |
| Arg | 340 | 230 | 148% | 408 | 389 | 105% | 748 | 619 | 121% |
| Asn | 133 | 204 | 65% | 264 | 344 | 77% | 397 | 548 | 72% |
| Asp | 161 | 230 | 70% | 237 | 389 | 61% | 398 | 619 | 64% |
| Cys | 190 | 80 | 238% | 474 | 135 | 351% | 664 | 214 | 310% |
| Gln | 215 | 279 | 77% | 425 | 472 | 90% | 640 | 750 | 85% |
| Glu | 295 | 181 | 163% | 382 | 307 | 124% | 677 | 488 | 139% |
| Gly | 270 | 314 | 86% | 515 | 531 | 97% | 785 | 846 | 93% |
| His | 232 | 97 | 239% | 420 | 165 | 255% | 652 | 262 | 249% |
| Ile | 141 | 243 | 58% | 281 | 412 | 68% | 422 | 655 | 64% |
| Leu | 230 | 403 | 57% | 481 | 681 | 71% | 711 | 1 084 | 66% |
| Lys | 314 | 257 | 122% | 308 | 434 | 71% | 622 | 691 | 90% |
| Met | 123 | 124 | 99% | 279 | 210 | 133% | 402 | 333 | 121% |
| Phe | 248 | 173 | 143% | 422 | 292 | 145% | 670 | 465 | 144% |
| Pro | 410 | 226 | 181% | 291 | 382 | 76% | 701 | 607 | 115% |
| Ser | 193 | 327 | 59% | 542 | 554 | 98% | 735 | 881 | 83% |
| Thr | 136 | 266 | 51% | 315 | 449 | 70% | 451 | 715 | 63% |
| Trp | 185 | 58 | 319% | 196 | 97 | 202% | 381 | 155 | 246% |
| Tyr | 251 | 146 | 172% | 384 | 247 | 155% | 635 | 393 | 162% |
| Val | 154 | 286 | 54% | 323 | 487 | 66% | 477 | 774 | 62% |
| All | 4,456 | 4,456 | | 7,538 | 7,538 | | 43,882 | 43,882 | |

[a] – Expected numbers of amino acid residues were extracted from 105,990 sequences in the non-redundant OWL protein database (release 26.0)
[b] – O/E ratio – observed to expected ratio
