# Supplementary material for: PredictSNP: Robust and Accurate Consensus Classifier for Prediction of Disease-Related Mutations
Source: PLoS Comput Biol. 2014 Jan 16;10(1):e1003440. doi: 10.1371/journal.pcbi.1003440 (PMC3894168; doi:10.1371/journal.pcbi.1003440)
Supplement: Table S4 — Composition of OVERFIT testing dataset. (PDF) [file pcbi.1003440.s010.pdf]

Table S4. Composition of OVERFIT dataset.

Amino acid residues	Wild-type								
	Pathogenic variants			Neutral variants			All variants		
	Observed	Expected [a]	O/E ratio [b]	Observed	Expected [a]	O/E ratio [b]	Observed	Expected [a]	O/E ratio [b]
Ala	1,095	1,319	83%	1,320	1,123	118%	2,415	2,442	99%
Arg	3,013	915	330%	1,937	779	249%	4,950	1,694	292%
Asn	526	808	65%	573	689	83%	1,099	1,497	73%
Asp	861	914	94%	645	779	83%	1,506	1,693	89%
Cys	954	316	302%	241	270	89%	1,195	586	204%
Gln	377	1,107	34%	540	943	57%	917	2,050	45%
Glu	758	720	105%	785	614	128%	1,543	1,334	116%
Gly	2,239	1,248	179%	870	1,063	82%	3,109	2,311	135%
His	463	387	120%	407	329	124%	870	716	122%
Ile	550	966	57%	816	824	99%	1,366	1,790	76%
Leu	1,285	1,599	80%	783	1,363	57%	2,068	2,962	70%
Lys	392	1,019	38%	509	869	59%	901	1,888	48%
Met	413	492	84%	516	419	123%	929	911	102%
Phe	455	685	66%	319	584	55%	774	1,269	61%
Pro	931	896	104%	986	764	129%	1,917	1,660	115%
Ser	944	1 300	73%	1,151	1,108	104%	2,095	2,408	87%
Thr	711	1 054	67%	1,058	899	118%	1,769	1,953	91%
Trp	361	228	158%	110	195	57%	471	423	111%
Tyr	556	580	96%	290	494	59%	846	1,074	79%
Val	811	1 142	71%	1,225	973	126%	2,036	2,115	96%
All	17,695	17,695		15,081	15,081		32,776	32,776	
**Amino acid residues**	**Mutant**								
	**Pathogenic variants**			**Neutral variants**			**All variants**		
	**Observed**	**Expected [a]**	**O/E ratio [b]**	**Observed**	**Expected [a]**	**O/E ratio [b]**	**Observed**	**Expected [a]**	**O/E ratio [b]**
Ala	437	1,319	75%	889	1 123	79%	1,326	2,442	54%
Arg	2,006	915	220%	1 140	779	146%	3,146	1,694	186%
Asn	602	808	74%	598	689	87%	1,200	1,497	80%
Asp	815	914	89%	503	779	65%	1,318	1,693	78%
Cys	1,252	316	396%	552	270	205%	1,804	586	308%
Gln	794	1,107	72%	772	943	82%	1,566	2,050	76%
Glu	683	720	95%	554	614	90%	1,237	1,334	93%
Gly	744	1,248	60%	666	1 063	63%	1,410	2,311	61%
His	909	387	235%	779	329	236%	1,688	716	236%
Ile	539	966	56%	881	824	107%	1,420	1,790	79%
Leu	1,111	1,599	69%	1 027	1 363	75%	2,138	2,962	72%
Lys	764	1,019	75%	609	869	70%	1,373	1,888	73%
Met	501	492	102%	716	419	171%	1,217	911	134%
Phe	549	685	80%	409	584	70%	958	1,269	75%
Pro	1,445	896	161%	593	764	78%	2,038	1,660	123%
Ser	1,369	1,300	105%	1 339	1 108	121%	2,708	2,408	112%
Thr	834	1,054	79%	1 155	899	129%	1,989	1,953	102%
Trp	674	228	295%	306	195	157%	980	423	232%
Tyr	629	580	108%	264	494	53%	893	1,074	83%
Val	1,038	1,142	91%	1 329	973	137%	2,367	2,115	112%
All	17,695	17,695		7,538	7,538		32,776	32,776	

[a] – Expected numbers of amino acid residues were extracted from 105,990 sequences in the non-redundant OWL protein database (release 26.0)
[b] – O/E ratio – observed to expected ratio
