# Supplementary material for: PredictSNP: Robust and Accurate Consensus Classifier for Prediction of Disease-Related Mutations
Source: PLoS Comput Biol. 2014 Jan 16;10(1):e1003440. doi: 10.1371/journal.pcbi.1003440 (PMC3894168; doi:10.1371/journal.pcbi.1003440)
Supplement: Table S5 — Performance of prediction tools with PredictSNP benchmark dataset. (PDF) [file pcbi.1003440.s011.pdf]

Table S5. Performance of prediction tools with PredictSNP benchmark dataset.

	MAPP	nsSNPAnalyzer	PANTHER	PhD-SNP	PPH-1	PPH-2	SIFT	SNAP	PredictSNP
**True positives**	14,580	7,225	5,392	13,741	12,491	16,025	15,357	11,324	14,666
**False negatives**	4,829	4,295	5,181	6,059	6,905	3,775	4,156	8,108	5,134
**True negatives**	12,828	2,016	10,377	19,196	17,225	14,281	15,230	18,229	18,131
**False positives**	6,307	1,149	3,026	4,886	6,722	9,798	7,867	5,827	5,951
**Total**	**38,544**	**14,685**	**23,976**	**43,882**	**43,343**	**43,879**	**42,610**	**43,488**	**43,882**
**Sensitivity** [a]	0.751	0.627	0.510	0.694	0.644	0.809	0.787	0.583	0.741
**Specificity** [a]	0.670	0.637	0.774	0.797	0.719	0.593	0.659	0.758	0.753
**Precision** [a]	0.695	0.633	0.693	0.774	0.696	0.665	0.698	0.706	0.750
**NPV** [a]	0.729	0.631	0.612	0.723	0.669	0.757	0.756	0.645	0.744
**Accuracy** [a]	**0.711**	**0.632**	**0.642**	**0.746**	**0.682**	**0.701**	**0.723**	**0.670**	**0.747**
**MCC** [a]	**0.423**	**0.264**	**0.295**	**0.494**	**0.364**	**0.412**	**0.450**	**0.346**	**0.494**
**AUC** [a]	**0.773**	**0.634**	**0.692**	**0.812**	**0.695**	**0.776**	**0.784**	**0.732**	**0.808**

PPH-1 – PolyPhen-1; PPH-2 – PolyPhen-2; NPV – negative predictive value; MCC – Matthews correlation coefficient; AUC – area under receiver operating characteristics curve; [a] – these metrics were calculated with normalized numbers
