# Supplementary material for: PredictSNP: Robust and Accurate Consensus Classifier for Prediction of Disease-Related Mutations
Source: PLoS Comput Biol. 2014 Jan 16;10(1):e1003440. doi: 10.1371/journal.pcbi.1003440 (PMC3894168; doi:10.1371/journal.pcbi.1003440)
Supplement: Table S6 — Performance of prediction tools with PMD testing dataset. (PDF) [file pcbi.1003440.s012.pdf]

Table S6. Performance of prediction tools with PMD testing dataset.

	MAPP	nsSNPAnalyzer	PANTHER	PhD-SNP	PPH-1	PPH-2	SIFT	SNAP	PredictSNP
**True positives**	1,353	892	390	1,521	1,468	1,846	1,379	1,345	1,738
**False negatives**	294	589	262	728	709	357	275	781	511
**True negatives**	576	482	478	735	773	526	480	756	688
**False positives**	613	253	202	513	445	710	579	444	560
**Total**	**2,836**	**2,216**	**1,332**	**3,497**	**3,395**	**3,439**	**2,713**	**3,326**	**3,497**
**Sensitivity** [a]	0.821	0.602	0.598	0.676	0.674	0.838	0.834	0.633	0.773
**Specificity** [a]	0.484	0.656	0.703	0.589	0.635	0.426	0.453	0.630	0.551
**Precision** [a]	0.614	0.636	0.668	0.622	0.649	0.593	0.604	0.631	0.633
**NPV** [a]	0.731	0.622	0.636	0.645	0.661	0.724	0.732	0.632	0.708
**Accuracy** [a]	**0.653**	**0.629**	**0.651**	**0.633**	**0.654**	**0.632**	**0.643**	**0.631**	**0.662**
**MCC** [a]	**0.327**	**0.258**	**0.303**	**0.266**	**0.309**	**0.288**	**0.310**	**0.263**	**0.332**
**AUC** [a]	**0.695**	**0.630**	**0.697**	**0.676**	**0.658**	**0.704**	**0.685**	**0.667**	**0.720**

PPH-1 – PolyPhen-1; PPH-2 – PolyPhen-2; NPV – negative predictive value; MCC – Matthews correlation coefficient; AUC – area under receiver operating characteristics curve; [a] – these metrics were calculated with normalized numbers
