# Supplementary material for: PredictSNP: Robust and Accurate Consensus Classifier for Prediction of Disease-Related Mutations
Source: PLoS Comput Biol. 2014 Jan 16;10(1):e1003440. doi: 10.1371/journal.pcbi.1003440 (PMC3894168; doi:10.1371/journal.pcbi.1003440)
Supplement: Table S7 — Performance of prediction tools with MMP testing dataset. (PDF) [file pcbi.1003440.s013.pdf]

Table S7. Performance of prediction tools with MMP testing dataset.

	MAPP	nsSNPAnalyzer	PANTHER	PhD-SNP	PPH-1	PPH-2	SIFT	SNAP	PredictSNP
**True positives**	3,337	2,510	829	3,399	3,330	3,769	3,675	3,163	3,773
**False negatives**	1,113	1,518	1,428	1,058	944	505	416	1,293	683
**True negatives**	4,990	4,264	4,336	3,739	4,390	3,518	2,887	5,338	4,291
**False positives**	2,530	2,687	834	3,798	3,053	3,925	4,463	2,200	3,247
**Total**	**11,970**	**10,979**	**7,427**	**11,994**	**11,717**	**11,717**	**11,441**	**11,994**	**11,994**
**Sensitivity** [a]	0.750	0.623	0.367	0.763	0.779	0.882	0.898	0.710	0.847
**Specificity** [a]	0.664	0.613	0.839	0.496	0.590	0.473	0.393	0.708	0.569
**Precision** [a]	0.690	0.617	0.695	0.602	0.655	0.626	0.597	0.709	0.663
**NPV** [a]	0.726	0.619	0.570	0.676	0.728	0.800	0.794	0.709	0.788
**Accuracy** [a]	**0.707**	**0.618**	**0.603**	**0.629**	**0.684**	**0.677**	**0.646**	**0.709**	**0.708**
**MCC** [a]	**0.415**	**0.237**	**0.234**	**0.268**	**0.376**	**0.389**	**0.337**	**0.418**	**0.433**
**AUC** [a]	**0.759**	**0.620**	**0.676**	**0.685**	**0.720**	**0.774**	**0.710**	**0.769**	**0.787**

PPH-1 – PolyPhen-1; PPH-2 – PolyPhen-2; NPV – negative predictive value; MCC – Matthews correlation coefficient; AUC – area under receiver operating characteristics curve; [a] – these metrics were calculated with normalized numbers
