# Supplementary material for: PredictSNP: Robust and Accurate Consensus Classifier for Prediction of Disease-Related Mutations
Source: PLoS Comput Biol. 2014 Jan 16;10(1):e1003440. doi: 10.1371/journal.pcbi.1003440 (PMC3894168; doi:10.1371/journal.pcbi.1003440)
Supplement: Table S8 — Performance of prediction tools with OVERFIT testing dataset. (PDF) [file pcbi.1003440.s014.pdf]

Table S8. Performance of prediction tools with OVERFIT testing dataset.

	MAPP	nsSNPAnalyzer	PANTHER	PhD-SNP	PPH-1	PPH-2	SIFT	SNAP	PredictSNP
**True positives**	8,738	7,635	5,584	14,837	12,500	15,609	14,079	8,157	14,747
**False negatives**	3,665	3,242	3,455	2,852	4,884	2,085	2,700	4,314	2,948
**True negatives**	10,355	1,977	8,484	13,292	11,189	9,729	9,905	11,963	12,261
**False positives**	2,482	1,804	2,2224	1,781	3,728	5,302	4,329	3,017	2,820
**Total**	**25,240**	**14,658**	**19,474**	**32,762**	**32,301**	**32,725**	**31,013**	**27,451**	**32,776**
**Sensitivity** [a]	0.705	0.702	0.618	0.839	0.719	0.882	0.839	0.654	0.833
**Specificity** [a]	0.807	0.523	0.792	0.161	0.750	0.647	0.696	0.799	0.813
**Precision** [a]	0.785	0.595	0.748	0.877	0.742	0.714	0.734	0.765	0.817
**NPV** [a]	0.732	0.637	0.675	0.845	0.728	0.846	0.812	0.698	0.830
**Accuracy** [a]	**0.756**	**0.612**	**0.705**	**0.860**	**0.735**	**0.765**	**0.767**	**0.726**	**0.823**
**MCC** [a]	**0.514**	**0.229**	**0.416**	**0.721**	**0.469**	**0.545**	**0.541**	**0.457**	**0.647**
**AUC** [a]	**0.823**	**0.614**	**0.779**	**0.926**	**0.733**	**0.849**	**0.835**	**0.798**	**0.889**
**Size of training dataset**	N/A [b]	4,013	N/A [b]	34,314	N/A [b]	12,392	N/A [b]	81,312	N/A [b]
**Overlap between OVERFIT dataset and the training dataset of a tool**	N/A [b]	3,456	N/A [b]	30,889	N/A [b]	5,329	N/A [b]	1,173	N/A [b]
**Part of OVERFIT dataset composed from the training dataset of a tool**	N/A [b]	10.5%	N/A [b]	94.2%	N/A [b]	16.3%	N/A [b]	3.6%	N/A [b]

PPH-1 – PolyPhen-1; PPH-2 – PolyPhen-2; NPV – negative predictive value; MCC – Matthews correlation coefficient; AUC – area under receiver operating characteristics curve; [a] – these metrics were calculated with normalized numbers; [b] – no training dataset available
