# Supplementary material for: PredictSNP: Robust and Accurate Consensus Classifier for Prediction of Disease-Related Mutations
Source: PLoS Comput Biol. 2014 Jan 16;10(1):e1003440. doi: 10.1371/journal.pcbi.1003440 (PMC3894168; doi:10.1371/journal.pcbi.1003440)
Supplement: Table S9 — Comparison of performance evaluation with Thusberg dataset and PredictSNP benchmark dataset. (PDF) [file pcbi.1003440.s015.pdf]

Table S9. Comparison of performance evaluation with Thusberg dataset and PredictSNP benchmark dataset.

	Testing dataset	MAPP	nsSNPAnalyzer	PANTHER	PhD-SNP	PPH1	PPH2	SIFT	SNAP	MutPred	SNPs&GO
**Percent of evaluated mutations**	Thusberg[a]	- [b]	23.2	59.3	98.7	99.1	99.3	86.3	80.8	90.8	92.9
	PredictSNP benchmark	87.8	33.5	54.6	100.0	98.8	100.0	97.1	99.1	- [c]	- [c]
	Difference	- [b]	-10.3	4.7	-1.3	0.3	-0.7	-10.8	-18.3	- [c]	- [c]
**Accuracy** [d]	Thusberg[a]	- [b]	0.600	0.760	0.710	0.700	0.690	0.650	0.720	0.810	0.820
	PredictSNP benchmark	0.711	0.632	0.642	0.746	0.682	0.701	0.723	0.670	- [c]	- [c]
	Difference	- [b]	-0.032	0.118	-0.036	0.018	-0.011	-0.073	0.050	- [c]	- [c]
**Matthews correlation coefficient** [d]	Thusberg[a]	- [b]	0.190	0.530	0.430	0.400	0.390	0.300	0.470	0.630	0.650
	PredictSNP benchmark	0.423	0.264	0.295	0.494	0.364	0.412	0.450	0.346	- [c]	- [c]
	Difference	- [b]	-0.074	0.235	-0.064	0.036	-0.022	-0.150	0.124	- [c]	- [c]
**Size of the training dataset of evaluated tool**		- [e]	4,013	- [e]	34,314	- [e]	12,392	- [e]	31,812	65,656	38,469
**Overlap between testing dataset and the training dataset of a tool**	Thusberg[a]	- [e]	2,791	- [e]	15,500	- [e]	3,222	- [e]	856	20,333	16,222
	PredictSNP benchmark	- [e]	0	- [e]	0	- [e]	0	- [e]	0	- [c]	- [c]
**Part of the testing dataset composed from the training dataset of tool**	Thusberg[a]	- [e]	6.9%	- [e]	38.3%	- [e]	8.0%	- [e]	2.1%	50.2%	40.0%
	PredictSNP benchmark	- [e]	0.0%	- [e]	0.0%	- [e]	0.0%	- [e]	0.0%	- [c]	- [c]

PPH-1 – PolyPhen-1; PPH-2 – PolyPhen-2; [a] – results of performance evaluation taken from Thusberg *et al.* [25]; [b] – this tool was not evaluated with Thusberg dataset; [c] – this tool was not evaluated with PredictSNP benchmark dataset [d] – these metrics were calculated with normalized numbers; [e] – no training dataset available
