# Supplementary material for: PredictSNP: Robust and Accurate Consensus Classifier for Prediction of Disease-Related Mutations
Source: PLoS Comput Biol. 2014 Jan 16;10(1):e1003440. doi: 10.1371/journal.pcbi.1003440 (PMC3894168; doi:10.1371/journal.pcbi.1003440)
Supplement: Table S10 — Pairwise correlation of integrated tools. (PDF) [file pcbi.1003440.s016.pdf]

Table S10. Pairwise correlation of integrated tools.

	MAPP	PhD-SNP	PPH1	PPH2	SIFT
**PhD-SNP**	0.54				
**PPH1**	0.48	0.48			
**PPH2**	0.51	0.49	0.55		
**SIFT**	0.59	0.55	0.56	0.62	
**SNAP**	0.47	0.47	0.54	0.49	0.53

PPH-1 – PolyPhen-1; PPH-2 – PolyPhen-2
