# Supplementary material for: PredictSNP: Robust and Accurate Consensus Classifier for Prediction of Disease-Related Mutations
Source: PLoS Comput Biol. 2014 Jan 16;10(1):e1003440. doi: 10.1371/journal.pcbi.1003440 (PMC3894168; doi:10.1371/journal.pcbi.1003440)
Supplement: Table S11 — Performance of selected machine learning methods with PredictSNP, PMD and MMP datasets. (PDF) [file pcbi.1003440.s017.pdf]

Table S11. Performance of selected machine learning methods with PredictSNP, PMD and MMP datasets.

	Dataset	Weighted majority vote	Naïve Bayes	Logistic regression	Neural network	Support vector machine	Nearest neighbor	Random forest
Accuracy [a]	PredictSNP	0.747	0.741	0.760	0.758	0.758	0.678	0.713
	PMD	0.662	0.747	0.652	0.652	0.656	0.592	0.642
	MMP	0.708	0.683	0.671	0.685	0.682	0.604	0.661
Matthews correlation coefficient [a]	PredictSNP	0.494	0.484	0.522	0.524	0.517	0.357	0.448
	PMD	0.332	0.308	0.306	0.304	0.317	0.184	0.286
	MMP	0.433	0.400	0.357	0.374	0.384	0.213	0.323

[a] – these metrics were calculated with normalized numbers
