# Supplementary material for: PredictSNP: Robust and Accurate Consensus Classifier for Prediction of Disease-Related Mutations
Source: PLoS Comput Biol. 2014 Jan 16;10(1):e1003440. doi: 10.1371/journal.pcbi.1003440 (PMC3894168; doi:10.1371/journal.pcbi.1003440)
Supplement: Table S12 — Performance of consensus classifiers with PMD-UNIPROT and MMP datasets. (PDF) [file pcbi.1003440.s018.pdf]

Table S12. Performance of consensus classifiers with PMD-UNIPROT and MMP datasets.

	PMD-UNIPROT			MMP		
	**CONDEL**	**Meta-SNP**	**PredictSNP**	**CONDEL**	**Meta-SNP**	**PredictSNP**
**True positives**	872	676	714	4,181	3,329	3,773
**False negatives**	40	236	198	276	1,119	683
**True negatives**	87	310	298	2,583	4,495	4,291
**False positives**	431	208	2220	4,954	3,015	3,247
**Total**	**1,430**	**1,430**	**1,430**	**11,994**	**11,958**	**11,994**
**Sensitivity** [a]	0.956	0.741	0.783	0.938	0.748	0.847
**Specificity** [a]	0.168	0.598	0.575	0.343	0.599	0.569
**Precision** [a]	0.535	0.649	0.648	0.588	0.651	0.663
**NPV** [a]	0.793	0.698	0.726	0.847	0.704	0.788
**Accuracy** [a]	**0.562**	**0.670**	**0.679**	**0.640**	**0.673**	**0.708**
**MCC** [a]	**0.202**	**0.343**	**0.366**	**0.349**	**0.351**	**0.433**
**AUC** [a]	**0.755**	**0.709**	**0.732**	**0.770**	**0.730**	**0.780**

NPV – negative predictive value; MCC – Matthews correlation coefficient; AUC – area under receiver operating characteristics curve; [a] – these metrics were calculated with normalized numbers
