# Supplementary material for: PredictSNP: Robust and Accurate Consensus Classifier for Prediction of Disease-Related Mutations
Source: PLoS Comput Biol. 2014 Jan 16;10(1):e1003440. doi: 10.1371/journal.pcbi.1003440 (PMC3894168; doi:10.1371/journal.pcbi.1003440)
Supplement: Table S13 — Performance of consensus classifiers PON-P and PredictSNP with PMD-UNIPROT and MMP datasets. (PDF) [file pcbi.1003440.s019.pdf]

Table S13. Performance of consensus classifiers PON-P and PredictSNP with PMD-UNIPROT and MMP datasets.

	PMD-UNIPROT			MMP		
	**PON-P**	**PredictSNP [a]**	**PredictSNP**	**PON-P**	**PredictSNP [a]**	**PredictSNP**
**True positives**	362	523	714	1,461	2,743	3,773
**False negatives**	192	89	198	875	258	683
**True negatives**	268	159	298	4,152	2,619	4,291
**False positives**	65	116	220	522	1,390	3,247
**Total**	**887**	**887**	**1,430**	**7,010**	**7,010**	**11,994**
**Sensitivity [b]**	0.653	0.855	0.783	0.625	0.914	0.847
**Specificity [b]**	0.805	0.578	0.575	0.888	0.653	0.569
**Precision [b]**	0.770	0.670	0.648	0.848	0.725	0.663
**NPV [b]**	0.699	0.799	0.726	0.703	0.884	0.788
**Accuracy [b]**	**0.729**	**0.716**	**0.679**	**0.757**	**0.784**	**0.708**
**MCC [b]**	**0.464**	**0.450**	**0.366**	**0.532**	**0.588**	**0.433**
**AUC [b]**	**0.724**	**0.740**	**0.724**	**0.790**	**0.804**	**0.787**

[a] – PredictSNP set up to return predictions for the same number of mutations as PON-P; these mutations have the highest PredictSNP score reflecting the degree of confidence in its decision; [b] – these metrics were calculated with normalized numbers
