# Supplementary material for: PredictSNP: Robust and Accurate Consensus Classifier for Prediction of Disease-Related Mutations
Source: PLoS Comput Biol. 2014 Jan 16;10(1):e1003440. doi: 10.1371/journal.pcbi.1003440 (PMC3894168; doi:10.1371/journal.pcbi.1003440)
Supplement: Text S2 — PredictSNP user guide. (PDF) [file pcbi.1003440.s021.pdf]

PredictSNP 1.0: Robust and Accurate Consensus Classifier for Prediction of Disease-Related Mutations

User guide

Contact:

Loschmidt Laboratories,

Department of Experimental Biology and Research Centre for Toxic Compounds in the Environment,

Faculty of Science, Masaryk University,

Kamenice 5, Bld. A13, 625 00 Brno, Czech Republic

Webpage: http://loschmidt.chemi.muni.cz

E-mail: predictsnp@gmail.com

Recommended Citation:

Bendl J., Stourac J., Salanda O., Pavelka A., Wieben E.D., Zendulka J., Brezovsky J. and Damborsky J. **PredictSNP: robust and accurate consensus classifier for prediction of disease-related mutations**. *Submitted for publication*.

© Copyright 2011-2013 Loschmidt Laboratories, Department of Experimental Biology and Research Centre for Toxic Compounds in the Environment, Faculty of Science, Masaryk University, Brno, Czech Republic

Contents

1.Introduction 5

2. Input page 6

3. Output page 9

4. Raw results 12

5. Right banner 13

6. Example 14

7. Standalone version 16

1. INTRODUCTION

Single nucleotide variants represent a prevalent form of genetic variation. Mutations in the coding regions are frequently associated with the development of various genetic diseases. Computational tools for the prediction of the effects of mutations on protein function are very important for analysis of single nucleotide variants and their prioritization for experimental characterization. Many computational tools are already widely employed for this purpose. The PredictSNP is a consensus classifier combining six best performing prediction methods to provide more accurate and robust alternative to the predictions delivered by individual integrated tools. The predictions from the computational tools are supplemented by experimental annotations from two databases. The web server is freely available to the academic community at http://loschmidt.chemi.muni.cz/predictsnp.

2. INPUT PAGE

The process of submitting the job to PredictSNP server consists of following steps:

1. **INPUT section** – paste an amino acid sequence of a query protein in FASTA format and press "Load" button. NOTE: the input sequence will be translated into interactive sequence into the section MUTATIONS.

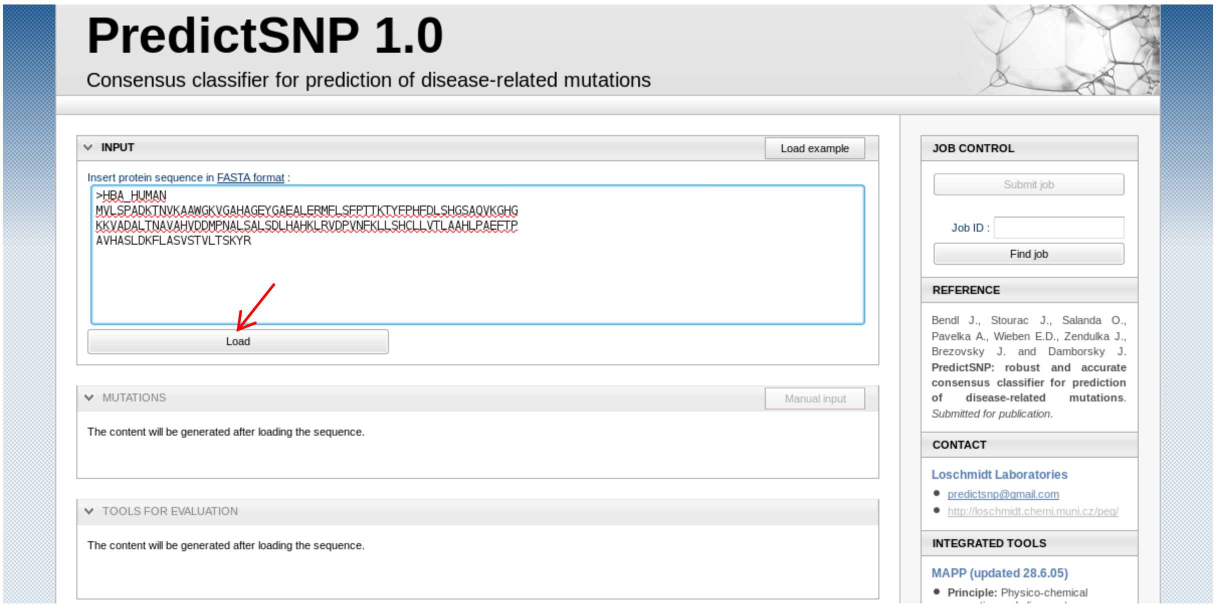

2. **MUTATIONS section**
   a. *select mutations using interactive sequence* – select position and then define required mutations, e.g., selection of wild-type Leucine at position 3, and mutant variants Cystein, Histidine and Asparagine. NOTE: To mutate the selected position to all variants, use option "ALL".

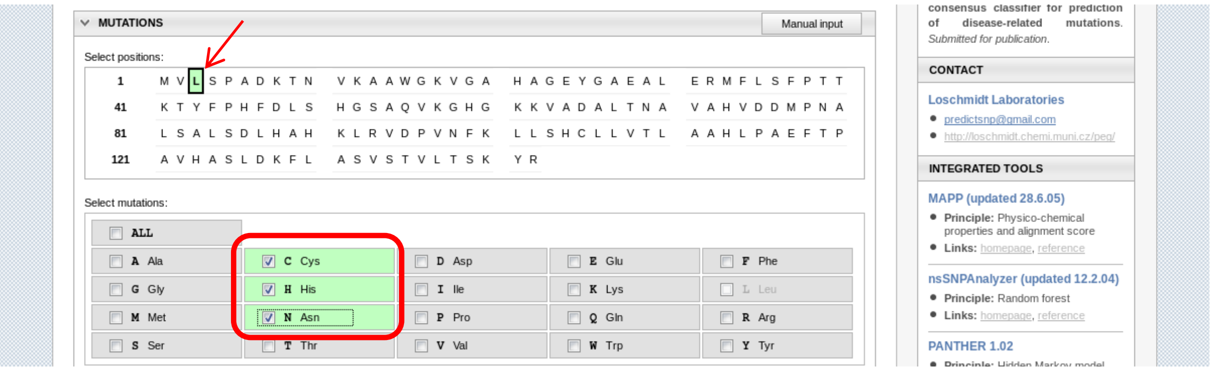

b. *submit a list of mutations in the text format* – press "Manual input" button. The required format of the text consists of one character abbreviation of wild-type residue, residue position, and one character abbreviation of mutant residue, e.g., L3C, L3H and L3N.

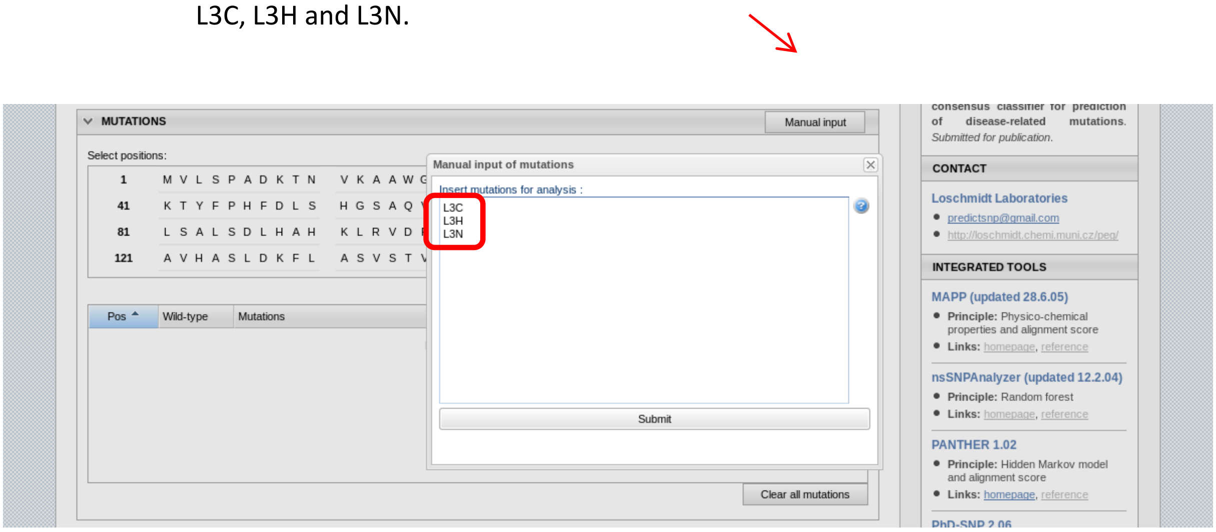

c. TIPS: You can change selected mutations by selecting the wild-type residue in the interactive sequence and then modifying the selection in the panel "Select mutations". Alternatively, you can remove all mutations from a single position by clicking on ⊖ symbol in the table of selected mutations, or remove all selected mutations by clicking on the button "Clear all mutations".

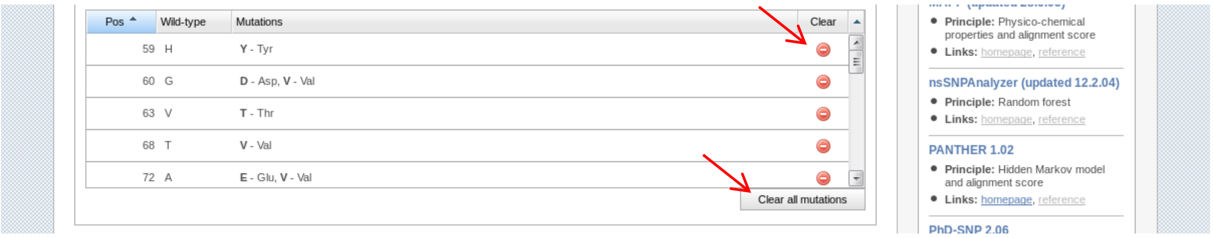

3. **TOOLS FOR EVALUATION** section – select tools to be employed for the evaluation of selected mutations. Time demands are estimated for each tool based on the average time of individual tools needed for evaluation of a given number of mutations. The information on the expected accuracy of the tools is also supplemented. Finally, the estimated waiting time of your job in the job queue is provided. NOTE: For the calculation of the PredictSNP consensus, all six constituent tools (MAPP, PhD-SNP, PolyPhen-1, PolyPhen-2, SIFT and SNAP) have to be selected.

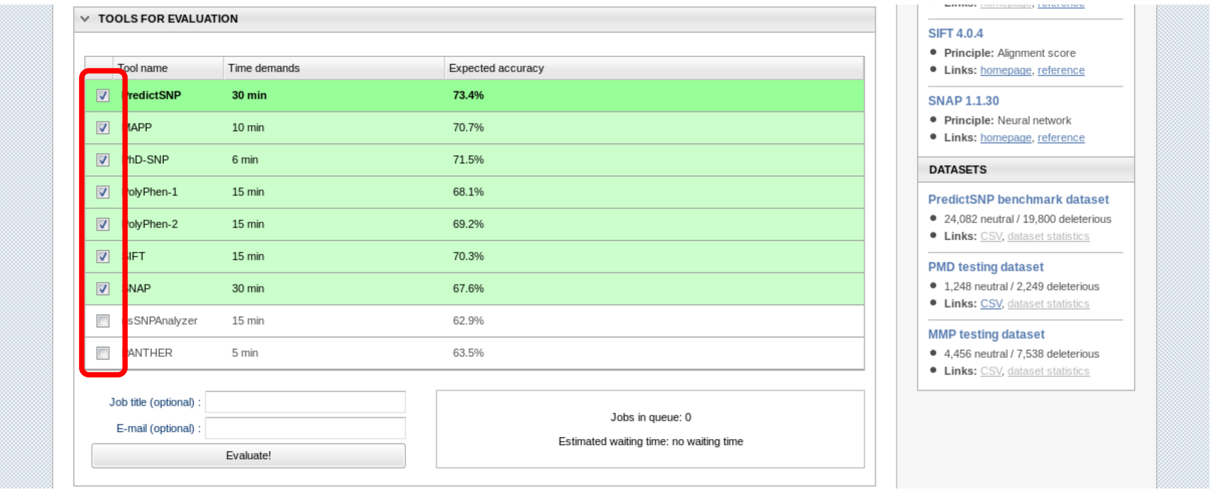

4. OPTIONALLY: Provide a job title and e-mail address on which the information about the job will be sent.

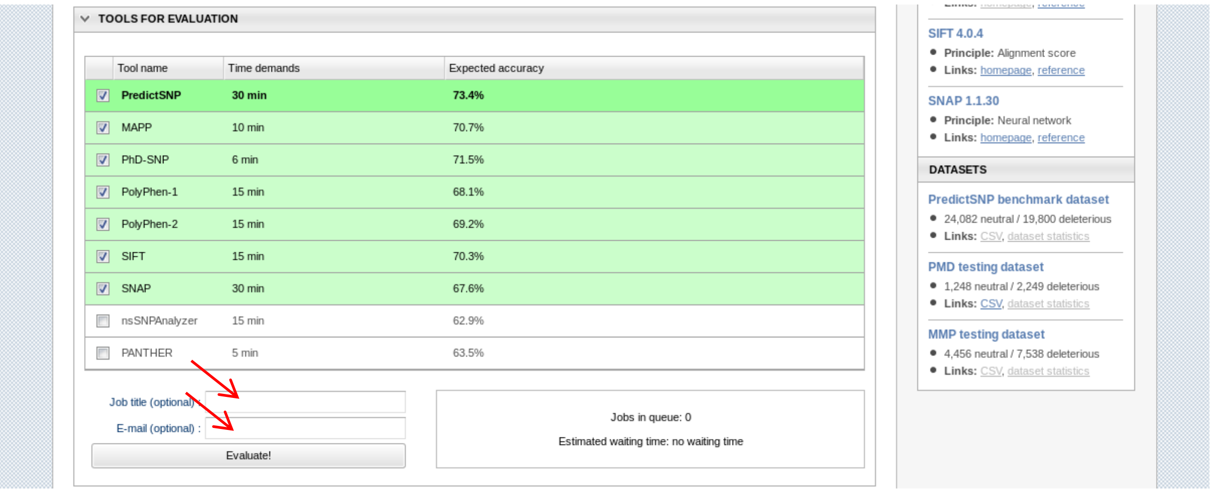

3. OUTPUT PAGE

Upon successful submission of a job, the user is redirected on the output page. The following information is available:

1. Job status – whether the job is running or waiting in the queue.

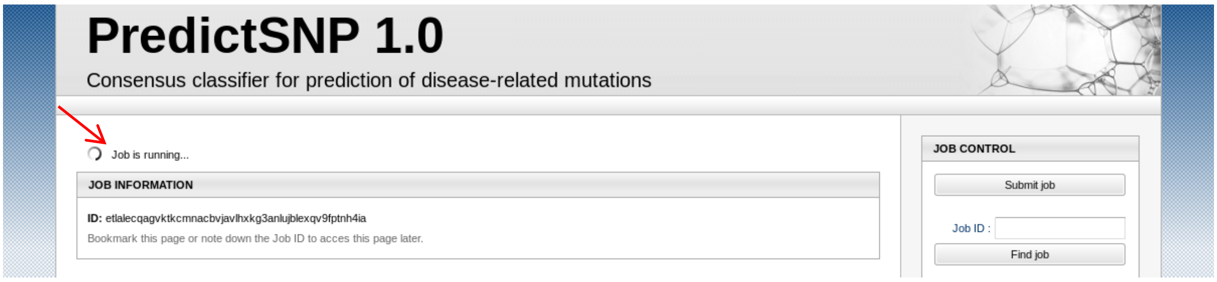

2. **JOB INFORMATION** section – provides information about Job ID which can be used to access the job via JOB CONTROL section of the right banner. NOTE: Alternatively, you can either bookmark whole page to access the job later, or provide an e-mail address on the input page.

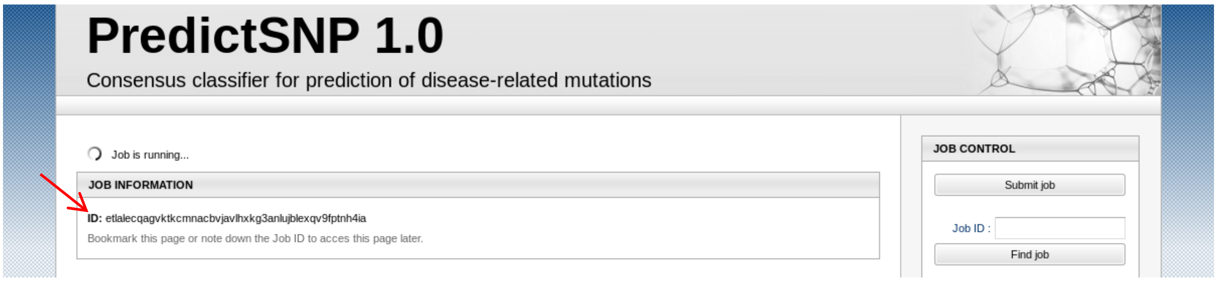

3. **LOG RECORDS** section – provides information on process of calculation including status of prediction of individual tools, construction of PredictSNP consensus and querying the databases.

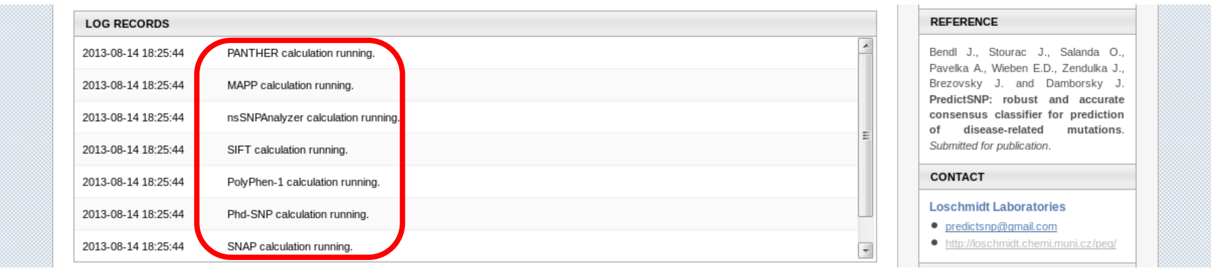

4. **RESULTS** section – once the job is finished, the prediction of each tool and PredictSNP is provided for all selected mutations. The predicted effect is color-coded: neutral mutations are in green, while deleterious mutations in red. The "-" symbol indicates that the respective mutation was not evaluated by a given tool. The normalized confidence of the tools is represented as a percentage. The ▸ symbol in the first column indicates that a relevant annotation was found in the database for a given mutation. By clicking on this symbol, users can show/hide the annotation. The annotations provide description of experimentally observed effects of a given mutation as well as the links to the original database records. NOTE: To show/hide all annotations at once use "Expand all annotations" or "Collapse all annotations" button.

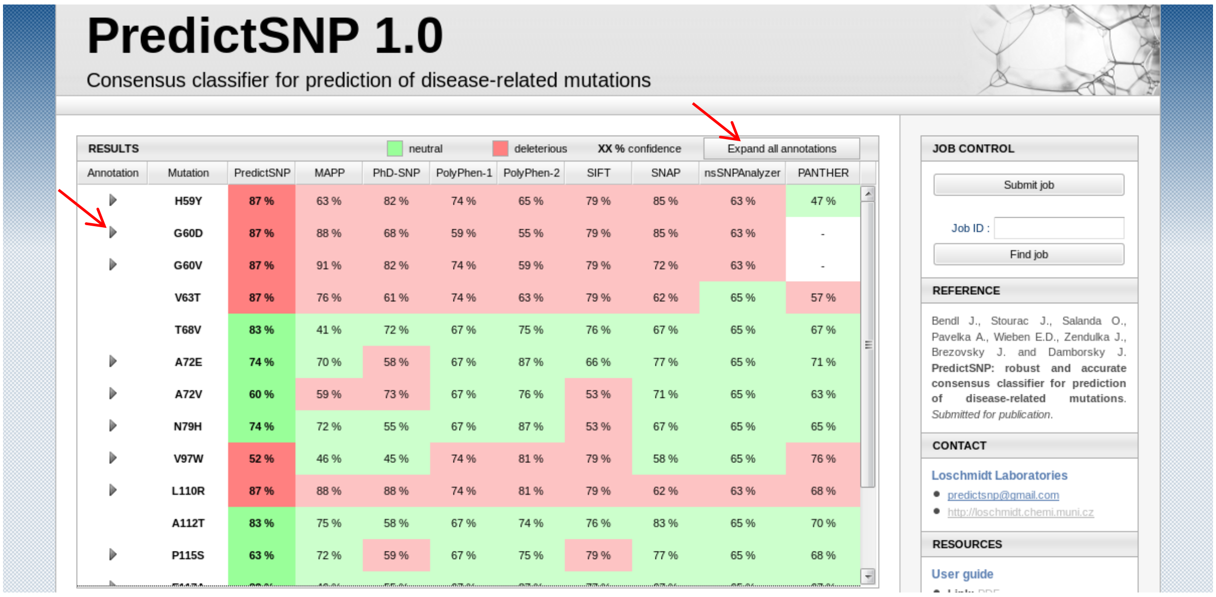

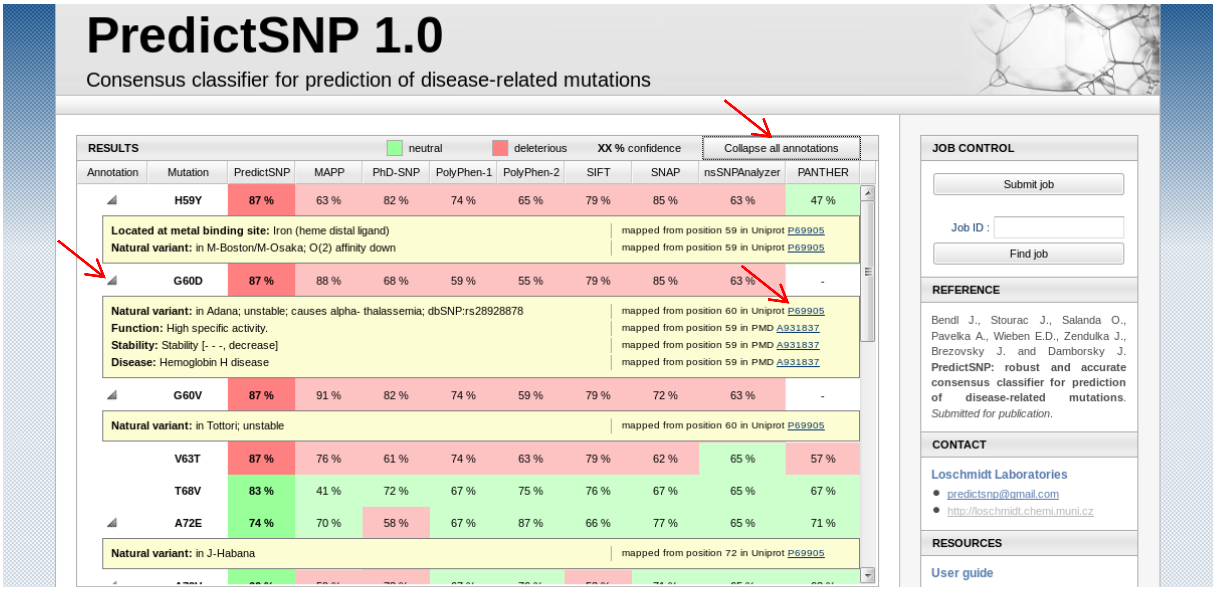

5. **DOWNLOAD** section – "Summary table" button enables to save the summary result table from **RESULTS** section in the form of CSV file. The "Raw results" button provides access to all files created during the calculations. NOTE: Detailed description of Raw results is provided in chapter 4 – Raw results.

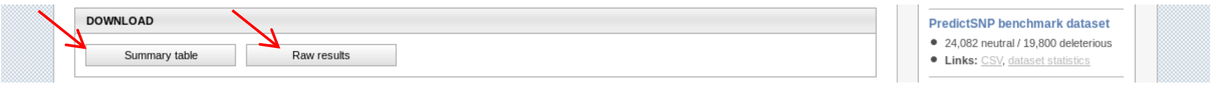

4. RAW RESULTS

The content of the zip package with results of a calculated job is described in the following table:

File	Description
job.conf	Input XML file with information about submitted job.
pathogenicity.csv	CSV file with summary result table from **RESULTS** section.
pathogenicity	Folder with intermediate results of individual prediction tools.
mapp.msa	Constructed multiple sequence alignment for MAPP prediction.
mapp.tree	Constructed phylogenetic tree for MAPP prediction.
mapp.out	Result of MAPP prediction.
nssnpanalyzer.out	Results of nsSNPAnalyzer prediction.
panther.out	Results of PANTHER prediction.
phdsnp.out	Results of PhD-SNP prediction.
polyphen.out	Results of PolyPhen-1 prediction.
polyphen2.out	Results of PolyPhen-2 prediction.
sift.out	Results of SIFT prediction.
snap.out	Results of SNAP prediction.

5. RIGHT BANNER

The right banner provides following information and services:

1. **JOB CONTROL** section – pressing "Submit job" button redirects the user to the INPUT page (see chapter 2) enabling submission of a new job. "Find job" button can be used to access an output page of a job with provided Job ID.
2. **REFERENCE** section – provides access to the article describing the methodology behind the PredictSNP consensus classifier.
3. **CONTACT** section – provides means to contact the developers.
4. **RESOURCES** section – enables download of this user guide, all constructed datasets and stand-alone version of PredictSNP consensus classifier.
5. **INTEGRATED TOOLS** section – provides links to web pages of all integrated tools as well as to the articles describing their development.

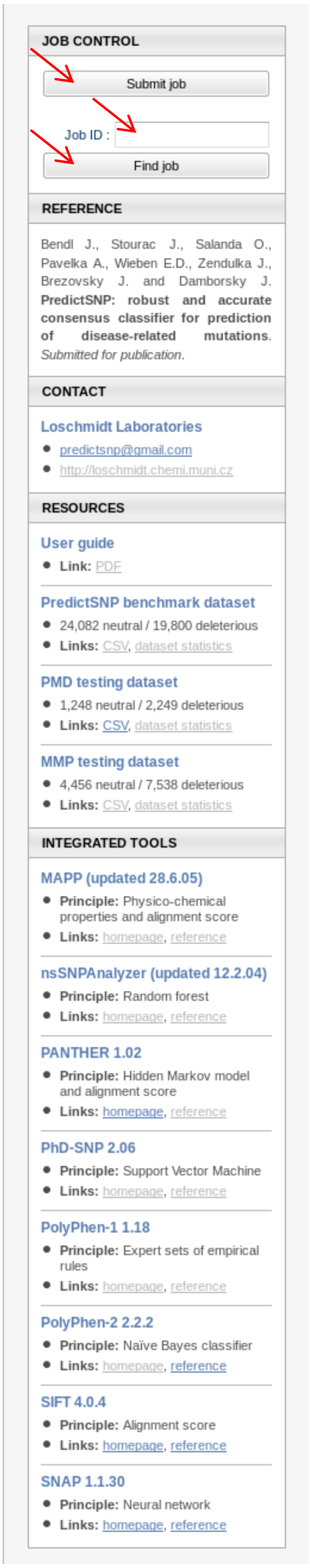

6. EXAMPLE

The PredictSNP server provides embedded example, which can be raised by pressing "Load example" button in the INPUT section at the input page. This loads the sequence of the alpha subunit of Human Hemoglobin and pre-selects 16 mutations at 13 positions.

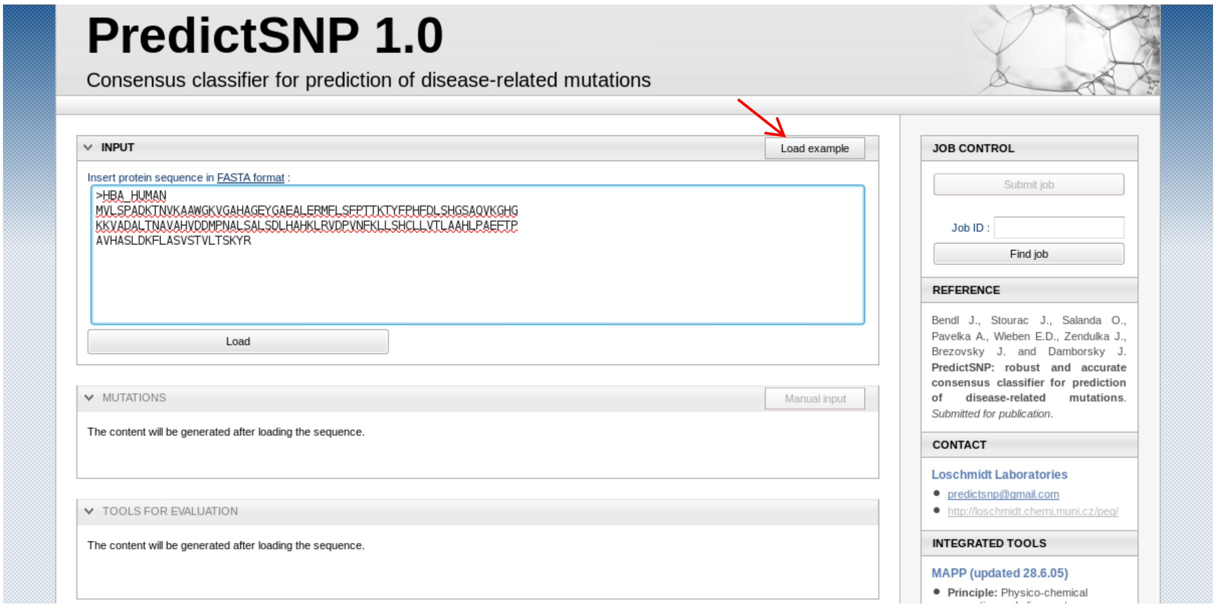

Once the evaluation of the mutations is finished, users can analyze the results in the result table.

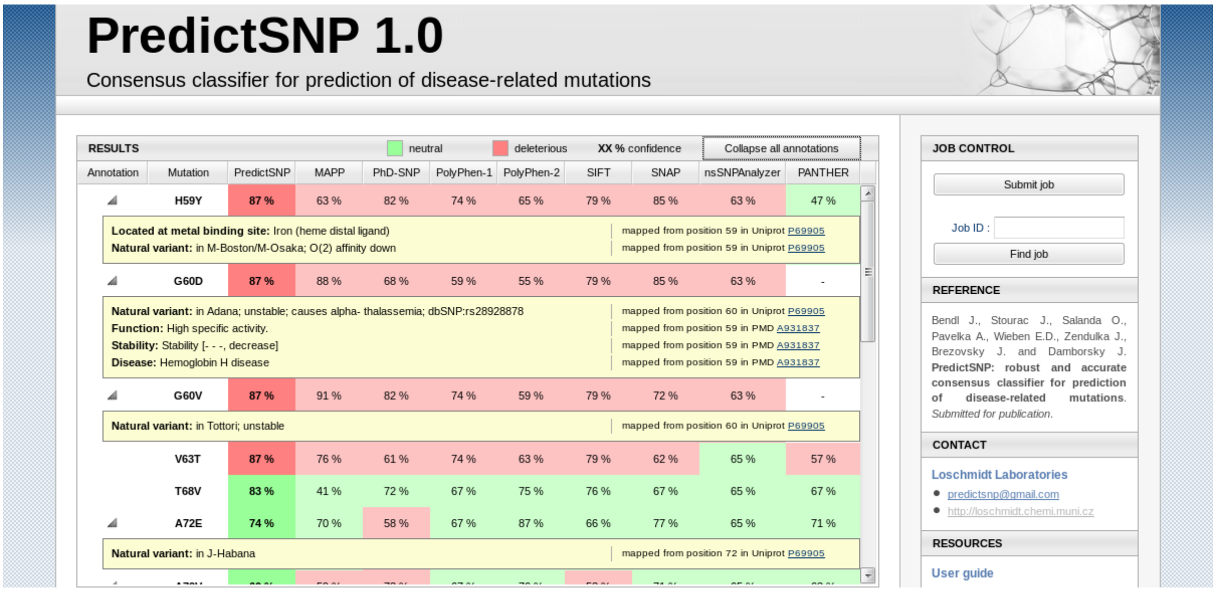

The results of such analysis provided by PredictSNP are summarized in following table:

Mutation	PredictSNP		Annotations	
	Effect	Confidence	PMD database	UniProt Database
H59Y	Deleterious	87%	-	Located at metal binding site<br>Natural variant: in M-Boston/M-Osaka<br>Oxygen affinity down
G60D	Deleterious	87%	High specific activity<br>Significant decreased stability<br>Disease: Hemoglobin H disease	Natural variant: in Adana<br>Protein unstable<br>Causes alpha-thalassemia
G60V	Deleterious	87%	-	Natural variant: in Tottori<br>Protein unstable
V63T	Deleterious	87%	-	-
T68V	Neutral	71%	-	-
A72E	Neutral	74%	-	Natural variant: in J-Habana
A72V	Neutral	60%	-	Natural variant: in Ozieri
N79H	Neutral	74%	-	Natural variant: in alpha-R, alpha-T, in alpha-2, in Davenport
V97W	Deleterious	61%	Decreased oxygen affinity<br>Increased cooperativity in oxygen binding	-
L110R	Deleterious	87%	-	Natural variant: in Suan-Dok;<br>Protein unstable<br>Causes alpha-thalassemia
A112T	Neutral	83%	-	-
P115S	Neutral	65%	-	Natural variant: in Melusine
E117A	Neutral	68%	-	Natural variant: in Ube-4
L126P	Deleterious	79%	-	Natural variant: in Quong Sze<br>Causes alpha-thalassemia
L126R	Deleterious	79%	-	Natural variant: in Plasencia<br>In family with moderate microcytosis and hypochromia
S132P	Deleterious	82%	-	Natural variant: in Questembert<br>Protein highly unstable<br>Causes alpha-thalassemia

As can be seen from the table, the results of prediction are frequently reflected by the experimental annotations. Mutations with deleterious effect correspond to natural variants with known clinical manifestation. This is in many cases accompanied by decreased stability of the protein. On the other hand, mutations predicted as neutral mostly correspond to natural variants without known negative effects. The mutations which are not annotated and at the same time are predicted with high confidence as deleterious, may represent interesting choice for experimental study. Conversely, the mutations predicted with high confidence as neutral should be deprioritized for further study.

7. STANDALONE VERSION

As alternative to the online version of PredictSNP consensus classifier, the standalone version suitable for massive mutagenesis studies is provided. In contrast to the online version of classifier, the standalone version requires pre-calculated predictions from all six integrated tools as input. For best performance, user should use the same version and settings of integrated tools as described in the Table 1 of Bendl et al. We recommend using the online version of classifier which is able to compute all required outputs of integrated tools. Moreover, the online version also provides experimental annotations from Protein Mutant Database and UniProt database.

Prerequisites:

Python (successfully tested on python2.6 and python2.7)

Installation:

1) Download and install prerequisites described above.

2) Unpack the distribution: *tar zxvf predictsnp-1.X.tar.gz*

Content of the archive:

predictsnp.py - Executable script.

predictsnp.data - Transformation functions of PredictSNP consensus.

testInput.txt - Input file for testing the PredictSNP consensus.

Usage:

$ python predictsnp.py [options]

Options:

-h, --help show help message and exit

-i, --input input file path; it contains confidence scores of integrated tools of PredictSNP (required)

-o, --output output file path (required)

Running PredictSNP standalone version with the test case:

Test case analyzes 16 mutations at 13 positions of the alpha subunit of Human Hemoglobin:

$ python predictsnp.py -i testInput.txt -o testOutput.txt

Format of the input file:

The analyzed mutations are placed on separate lines, where predictions from individual integrated tools are tab-delimited. All inputs are required; in case of missing prediction for some tool, put '**?**' instead. The example of the input file follows:

```
# Identifier  # Mutation  # MAPP  # PhD-SNP # PolyPhen-1  # PolyPhen-2  # SIFT  # SNAP
HBA_HUMAN H59Y  0.002402  Disease,6  probably damaging  0.997 DELETERIOUS,0 Non-neutral,4
HBA_HUMAN G60D  7.532E-007  Disease,3  possibly damaging  0.969 DELETERIOUS,0 Non-neutral,4
HBA_HUMAN G60V  0.000002485 Disease,6  probably damaging  0.988 DELETERIOUS,0.01  Non-neutral,2
HBA_HUMAN V63T  0.0007419 Disease,2  probably damaging  0.996 DELETERIOUS,0 Non-neutral,1
HBA_HUMAN T68V  0.09862 Neutral,6          benign  0.003 DELETERIOUS,0.02  Neutral,4
HBA_HUMAN A72E  0.3257  Disease,0          benign  0 TOLERATED,0.07  Neutral,6
HBA_HUMAN A72V  0.004843  Disease,4          benign  0.002 DELETERIOUS,0.01  Neutral,5
HBA_HUMAN N79H  0.441 Neutral,2          benign  0 DELETERIOUS,0.02  Neutral,4
HBA_HUMAN V97W  0.05  Neutral,0  probably damaging  1 DELETERIOUS,0 Neutral,2
HBA_HUMAN L110R 8.575E-007  Disease,8  probably damaging  1 DELETERIOUS,0 Non-neutral,1
HBA_HUMAN A112T 0.6192  Neutral,3          benign  0.006 TOLERATED,0.12  Neutral,8
HBA_HUMAN P115S 0.4512  Disease,1          benign  0.004 DELETERIOUS,0.02  Neutral,6
HBA_HUMAN E117A 0.05127 Neutral,2          benign  0 DELETERIOUS,0.05  Neutral,4
HBA_HUMAN L126P 0.00009128  Disease,6  probably damaging  1 DELETERIOUS,0 Neutral,0
HBA_HUMAN L126R 0.000001035 Disease,7  probably damaging  0.999 DELETERIOUS,0 Neutral,0
HBA_HUMAN S132P 0.02969 Disease,5  possibly damaging  0.789 DELETERIOUS,0 Neutral,4
```

Header	Explanation	Format	Example
# Identifier	Only for easy identification of mutation in the output file	String	HBA_HUMAN
# Mutation	Only for easy identification of mutation in the output file	String	H59Y
# MAPP	MAPP score	<0.0 - 1.0>	0.002402
# PhD-SNP	Prediction and reliability index (separated by comma)	<Disease | Neutral>,<0 - 9>	Disease,6
# Polyphen-1	Prediction	<probably damaging | possibly damaging | benign>	probably damaging
# Polyphen-2	Probability of the mutation being deleterious	<0.0 - 1.0>	0.997
# SIFT	SIFT score	<0.0 - 1.0>	0.01
# SNAP	Prediction and reliability index (separated by comma)	<Non-neutral | Neutral>,<0 - 9>	Non-neutral,4

Format of the output file:

The evaluated mutations are placed on separate lines in the same order as in the input file and with identical values of columns Identifier & Mutation. The predictions of PredictSNP consensus and individual tools are provided as well as their expected accuracies in the range between 0-100%.
